# Dynamic color change in the grouper *Variola louti* during interspecific interactions and swimming

**DOI:** 10.1093/beheco/araf005

**Published:** 2025-01-20

**Authors:** Sagi Marom, Moshe Kiflawi, Derya Akkaynak, Roi Holzman

**Affiliations:** School of Zoology, Faculty of Life Sciences, Tel Aviv University, P.O. Box 39040, Tel Aviv 6997801, Israel; The Inter-University Institute for Marine Sciences, P.O. Box 469, Eilat 88103, Israel; Department of Life Sciences, Ben Gurion University, P.O. Box 653, Beer Sheva 8410501, Israel; Hatter Department of Marine Technologies, Leon H. Charney School of Marine Sciences, University of Haifa, P.O. Box 3338, Haifa 3103301, Israel; School of Zoology, Faculty of Life Sciences, Tel Aviv University, P.O. Box 39040, Tel Aviv 6997801, Israel; The Inter-University Institute for Marine Sciences, P.O. Box 469, Eilat 88103, Israel; Steinhardt Museum of Natural History, Tel Aviv University, P.O. Box 39040, Tel Aviv 6997801, Israel

**Keywords:** agonistic signals, background matching, camouflage, cooperation, disruptive coloration, interspecific communication, fish, *Gymnothorax griseus*, *Gymnothorax nudivomer*, Serranidae

## Abstract

Animals can change their body color for various ecological functions. In fish, rapid dynamic color change is primarily known in contexts of intraspecific communication and camouflage, while examples in interspecific contexts are rare. We studied dynamic color changes and their associated behaviors in the grouper *Variola louti* in its native coral reef environment in the Red Sea. Using underwater videos to record natural behaviors and color-calibrated still images to measure body colors, we quantified color displays as the brightness of the body and the contrast of three distinct patterns: body patches, head stripe, and side bars. *V. louti* exhibited a diverse range of pattern displays, which rapidly transformed according to its behavioral shifts. A high-contrast head stripe pattern was observed when *V. louti* engaged in agonistic interspecific interactions, but was interestingly absent when hunting alone or in cooperation with moray eels. The brightness of *V. louti’s* body color and the contrasts of the body patches and side bars were associated with its swimming behavior. Darker body colors and high contrast body patches and side bars were expressed when the fish rested on the bottom, whereas bright and uniform body colors were displayed when swimming higher above the reef. Our results suggest that *V. louti* utilizes dynamic color displays for camouflage and interspecific communication in agonistic and competitive interspecific interactions. These findings highlight the importance of dynamic color changes for communication and provide valuable insights into the behavioral ecology of animals.

## Teaser

The grouper *Variola louti* is a fish that rapidly changes colors when interacting with other species. This behaviour is interesting as color change is seldom used for communication between species. We showed that the groupers change several independent patterns on their body to form different displays during specific behaviors. Some color patterns are potentially used for camouflage, while others seem to be expressed as aggressive signals for communication with other predatory species.

## Introduction

Animals feature a staggering diversity of body colors and patterns on their bodies (eg skin, scales, cuticle, feathers, and fur). Body color plays a role in thermoregulation (Forsman 2000; Smith et al. 2016; Hoppe 2018), photoprotection (Luecke and O’Brien 1981; Rózanowska et al. 1999; Herrling et al. 2008), courtship and mate choice (Grether et al. 2005), camouflage (Ramachandran et al. 1996; Stuart-Fox et al. 2006; Hanlon 2007; Stuart-Fox and Moussalli 2009; Watson et al. 2014; Duarte et al. 2017; Kelley et al. 2017; Pembury Smith and Ruxton 2020), mimicry (Hasson 1994), and warning (Kohda and Watanabe 1982; Muske and Fernald 1987; Korzan and Fernald 2007; Dijkstra et al. 2008); among other functions (Cuthill et al. 2017). While some animals retain the same colors and patterns throughout their life, others undergo color changes. Changes in coloration may occur slowly, eg ontologically (Cardwell and Liley 1991; Geissmann 1991; Crook 1997), or seasonally (Zimova et al. 2018; Giska et al. 2019), through changes in the concentrations of chromatophores and the pigments within them, a process known as “morphological color change” (Sugimoto 2002; Nordlund et al. 2008; Leclercq et al. 2010; Sköld et al. 2013, 2016). Alternatively, colors can change rapidly through aggregation and dispersal of pigments within chromatophore cells (Aspengren et al. 2008; Sköld et al. 2013, 2016) or alteration of the distance between reflective crystals (Mäthger et al. 2003; Gur et al. 2015), a process known as “physiological color change.” The ability of animals to rapidly modify the information conveyed via color displays provides high adaptability in changing circumstances. Appropriately, rapid dynamic color change is used to communicate warnings, emotions, and intentions (Batabyal and Thaker 2017; Caro and Allen 2017; Heathcote et al. 2018), as well as for context-dependent camouflage (Hanlon 2007; Hanlon et al. 2008) and mimicry (Norman et al. 2001). Although less common than morphological color change, rapid color change is phylogenetically widespread across invertebrates (Hinton and Jarman 1972; Hanlon 2007) and vertebrates, including reptiles (Stuart-Fox and Moussalli 2009; Ligon and McGraw 2013), amphibians (King et al. 1994; Kindermann et al. 2014), and fishes (Hamilton and Peterman 1971; Mäthger et al. 2003; Côté and Cheney 2005; Randall 2005; Cheney et al. 2008; Watson et al. 2014; Heathcote et al. 2018, 2020).

In fish, dynamic color changes are mostly known in the context of courtship displays (Morris et al. 1995; Fuller and Berglund 1996; Kodric-Brown 1998; Sköld et al. 2008), camouflage (Ramachandran et al. 1996; Akkaynak et al. 2017; Smithers et al. 2018), mimicry (Randall 2005), and other instances of intraspecific signalling (Haimo and Thaler 1994; Sköld et al. 2013, 2016; Heathcote et al. 2018). For example, Burns et al. (2024) recently found that striped marlin intensify the contrast of stripes on their body when attacking schools of prey fish, which was suggested to act as a signal to conspecifics for coordinating attacks and disrupting the evasion behavior of the schooling prey. In interspecific contexts, dynamic color changes are mostly associated with camouflage and mimicry, and are rarely found in other interspecific contexts (but see example in Heathcote et al. 2020). In Serranids, dynamic color changes have been documented in the context of camouflage (Erisman and Allen 2005; Watson et al. 2014), in intraspecific reproductive behaviors such as courtship and spawning (Clark 1959; Barlow 1975; Gilmore and Jones 1992; Erisman and Allen 2005), and antagonistic interactions during competition for mates (Kohda and Watanabe 1982). However, dynamic color changes in Serranids have not been previously documented in contexts of interspecific agonistic and cooperative behaviors.

**Fig. 1.**
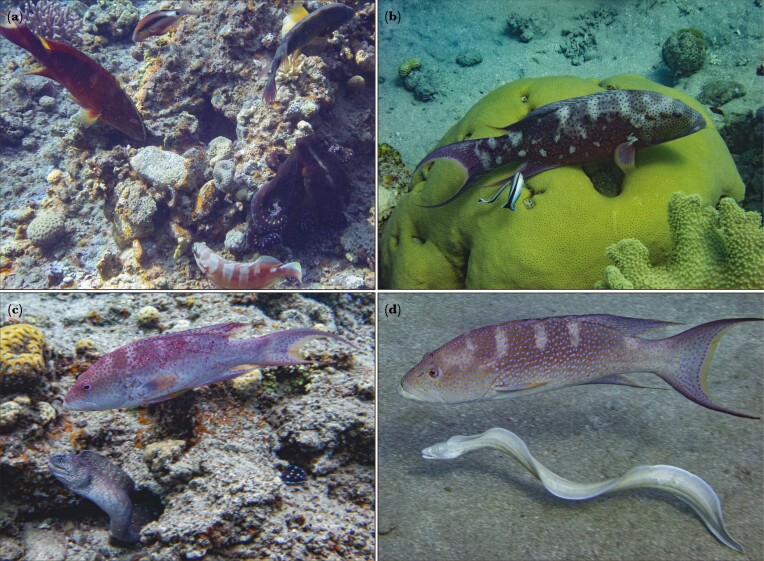
*Variola louti* engaging in interspecific interactions including agonistic (a), mutualistic (b), and cooperative (c–d), at the coral reef in the Gulf of Eilat, Red Sea. (a) *V. louti* hunting in association with other predators (*Epinephelus fasciatus*, *Parupeneus macronemus*, *Cheilinus lunulatus*, *Octopus cyanea*), (b) being cleaned by cleaner wrasses (*Labroides dimidiatus*), and (c–d) hunting in cooperation with moray eels (*Gymnothorax nudivomer* and *Gymnothorax griseus*, respectively).

**Fig. 2.**
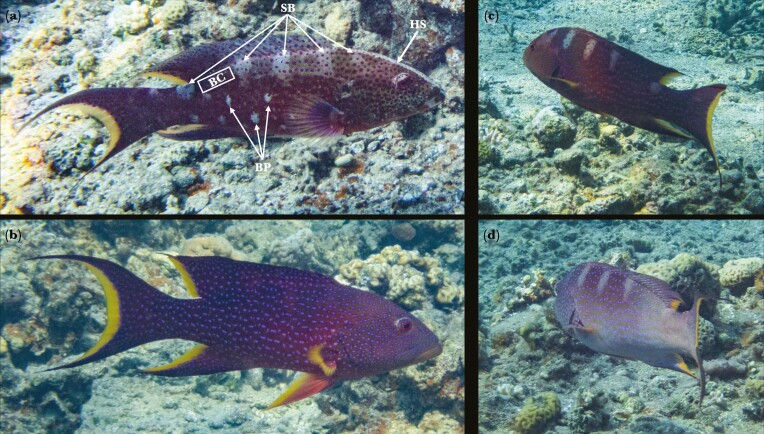
A single individual of *Variola louti* exhibiting rapid dynamic color change. The elapsed time between each image pair (a–b, c–d) is 3 ± 1 s. (a) expressing all three patterns very distinctly, (b) expressing none of the patterns with a dark body color, (c) expressing only the side bars with a dark body color, (d) expressing only the side bars with a bright body color. After color standardization (Fig. 3), the body color was measured in the area denoted [BC], side bars are denoted [SB], body patches are denoted [BP], and the head stripe is denoted [HS].

Here, we provide observations and quantitative evidence of dynamic color changes in the grouper *Variola louti* as it roamed its natural coral-reef habitat (Video S1). We documented fish during uninterrupted natural behaviors, including being cleaned by cleaner wrasses, hunting alone, hunting in multispecies groups, hunting in cooperation with moray eels, and interacting aggressively with other predators (Fig. 1). We asked whether dynamic color changes occur in *V. louti*, within what timeframes, and whether specific color patterns are associated with certain behaviors. We hypothesized that interspecific behaviors of *V. louti* are accompanied by distinct displays. Similarly, we hypothesized that changes in the fish’s movement patterns will be accompanied by color pattern variations. We used a video camera to record and classify the behaviors of the fish while simultaneously measuring their body colors using a still camera. By extracting the red, green, and blue pixel intensities from color-standardized images, we quantified the color displays as the brightness of the body and the contrast of three distinct patterns: body patches, side bars, and head stripe (Fig. 2).

## Methods

### Model organism

The Yellow-Edged Lyretail Grouper (*Variola louti,* Serranidae) is a predatory reef-associated species with a wide Indo-Pacific distribution. It is frequently observed at shallow depths (1 to 30 m), feeds primarily on fish and benthic crustaceans, and can reach a total length of ~80 cm and a maximum weight of 12 kg (Randall and Brock 1960; Harmelin-Vivien and Bouchon 1976; Fishelson 1977; Heemstra and Randall 1993; IGFA 2001; Lieske and Myers 2004; Nair et al. 2018; Binohlan and Capuli n.d.).

### In-situ image and video acquisition

SCUBA divers recorded video footage of naturally roaming fish at the Gulf of Eilat, Red Sea, using a GoPro Hero 3+ camera, while still photos were captured using an Olympus Tough tg-6 camera.

**Fig. 3.**
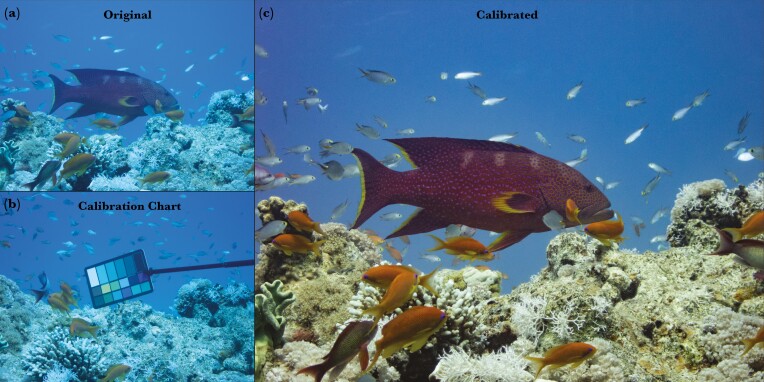
Image color standardization by white balancing. The original image (a) and an image of the color chart (b) with known RGB values subsequently positioned at the original location of the fish, were used for generating a color-calibrated image (c).

The video camera was mounted on top of the still camera and filmed continuously, while still photos were captured when *V. louti* was observed to change behavior, color, or both. The wide-angle view of the GoPro camera provided information on the behavioral context, while the still photographs were zoomed-in and focused on the body of the fish. After photographing the fish, a color chart was captured under the same light conditions and distance, serving as a reference for white balancing by RGB equalization (Akkaynak et al. 2014). This was done by placing the color chart at the position previously occupied by the fish immediately after it left the scene, while the diver operating the camera remained in place (Fig. 3). This procedure was performed after each photo sequence, defined as consecutive photos captured at a similar distance, depth, and direction within a brief time-frame (several seconds if the fish moved, and up to 1 min if the fish was stationary).

Observations were carried out in 30 dives. Although at each dive we were able to distinguish individual fish and note them as such, we could not determine whether the same fish was observed at different dives. However, because at least several hours (usually more than a day) elapsed between dives, we treated events from different dives as independent samples from different individuals. Thus, our sample size comprises 255 observations from 93 independent events.

### Image processing

Body color information was extracted from the images after verifying that the sensor of the still camera had a linear response to radiance using the methodology described and toolbox provided by Akkaynak et al. (2014). All still images were captured in RAW format (ORF files as produced by the Olympus camera), and pixel intensities were measured from color-standardized RAW still images in the device-specific RGB color space of the camera.

In an underwater setting, the optical properties of the water and the particles dissolved or suspended in it constitute a challenge for imaging, as they distort and occlude the “true” colors of subjects. Specifically, water and its constituents absorb and scatter light of different wavelengths at different rates in a distance-dependent manner (Morrison 1970; Buiteveld et al. 1994; Mobley 1995; Morel et al. 2007). This impairs the ability to compare the colors of subjects photographed at different depths, from different distances, or from different viewing angles. In addition, variations in ambient light (eg location, time of day, day of the year, cloud cover, etc.) and imaging geometry (ie the position of the camera and the subject relative to the sun), greatly influence the RGB values captured in a photograph. Thus, to meaningfully compare the colors of a subject across different photographs, the images must be acquired in a way that allows a computational removal of the degrading effects of water in a consistent manner. To design our image acquisition workflow for consistent color capture, we studied the underwater image formation model given by (Akkaynak and Treibitz 2018).

Briefly, underwater image formation is given by an equation that describes the dependence of captured RGB intensities to the imaging distance z (along line of sight) and optical parameters of the physical medium, namely the beam attenuation coefficient c(λ) and the diffuse downwelling attenuation coefficient $K_{d}\left(\lambda \right)$ (Akkaynak and Treibitz 2018). In this context, the distance z is pixelwise, meaning a distance map must be obtained for all pixels in an image; this is currently not possible to achieve underwater in real-time using a single camera. Commonly, pixelwise distance for underwater scenes is obtained in post-processing from multiple images taken by a single camera (Akkaynak and Treibitz 2019; but see eg Berman et al. 2021, when estimation from a single image pair is possible when multiple cameras are used in sync). However, both methods perform poorly when used to estimate the distance to moving objects such as fish. Thus, for our purposes, it was crucial to eliminate the requirement to obtain imaging distance z. Accordingly, we took all of our images from a close distance (ie z→0). In turn, the image formation equation simplifies greatly, and color standardization reduces to an easily solved problem of global white balancing, without the need to perform complex pixelwise operations (Akkaynak and Treibitz 2019).

Global white balancing is an operation which ensures that a surface that is neutral in color, like a gray surface, appears as a true gray in an image. This means that the pixel intensities for the Red (R), Green (G), and Blue (B) channels of the neutral surface should be equal. A “gray” surface is any surface with a spectrally uniform reflectance spectrum, while “white” is the brightest possible gray, with pixel values of R = G = B = 255 (in an 8-bit image). Thus, white balancing is achieved by extracting the RGB values of a gray target in an image and adjusting them to be equal. This operation removes global color casts from an image, making the image appear as if it was taken under white light.

In all our images, we used the same waterproof color target (DGK Color Tools WDKK; Fig. 3b and Fig. S1). This chart does not come factory-calibrated, so we measured the spectral reflectance of each of its patches in the lab.

Three important points should be noted here: (1) The color correction done through white balancing only applies to the objects at the distance where the color chart was placed, ie the distance at which the fish was from the camera. Meaning the color correction does not apply to other parts of the image (eg the reef background or other objects/organisms). (2) Since we always used the same camera to capture our images, we carried out all color-related analyses in the device-dependent RGB color space of our camera. This means that RGB values in our datasets (after color standardization) are comparable to one another, but without further processing, cannot be readily compared to RGB values that would be recorded by other cameras. (3) The calibrated RGB values are linear, and so when images are displayed (eg as seen in Figs. 1, 2, and 3c), they will appear to have low contrast and under-saturated colors. This is typical of linear RGB values prior to non-linear color enhancements are applied (akin to those performed by in-camera processing), and the images will therefore not have the vivid colors and contrast we are used to seeing from everyday photographs.

To efficiently process the images and analyze the color variables and behavior, we wrote a task-specific code in MATLAB 2019b version 9.7.0.1190202, which consists of four main parts:

(1) **Metadata extraction**

For compatibility reasons, all images were first converted from the RAW ORF format files into an equivalent RAW DNG format (linear demosaiced, uncompressed) using Adobe DNG Converter version 13.1.0.658. Then the RAW DNG image was loaded into the analysis code and the metadata (date, time, name, path, etc.) were extracted from the file.

(2) **Behavior analysis from videos**

Using the corresponding videos, the behavioral context of the image was evaluated according to three parameters:

(1) Activity—comprised the following six behavioral categories: no defined activity (none), hunting alone, hunting in cooperation with moray eels (*Gymnothorax griseus* and *Gymnothorax nudivomer*), being cleaned by cleaner wrasses (*Labroides dimidiatus*), hunting in association with other species, and aggression towards other predators. We observed *V. louti* hunting with or showing aggression toward *Cephalopholis miniata*, *Epinephelus tauvina*, *Epinephelus fasciatus*, *Aethaloperca rogaa*, *Parupeneus cyclostomus*, *Pterois miles*, and *Octopus cyanea*.
(2) Speed—comprised a qualitative assessment of the swimming speed of the fish (still/slow/medium/fast). Still was defined when the fish was stationary; slow as when the fish was moving <<1 body length per second; medium as normal cruising speed; and fast as swimming faster than a diver (ca. >>2 m·s^-^^1^).
(3) Distance from the bottom—was assigned one of two categories: closely adjacent to the bottom or positioned far above it (>1 m).

(3) **Color standardization**

This part of the code was adapted from Akkaynak et al. (2014). First, the photo containing the color chart was loaded and the 18% gray color pallet was marked (Fig. S1). The RGB values were extracted and averaged for each channel within the patch to obtain the RGB triplet to be used for white-balancing. Then, the corresponding image of the fish was white-balanced using those extracted RGB values (Fig. 3). All calibrated images were visually inspected. Fewer than 10 images presented exaggerated or unnatural colors after white-balancing and were excluded from the analysis. We discuss the sources of the error in the “Error estimation” section.

(4) **Color and pattern analysis from images**

Based on our observations of naturally behaved *V. louti*, we identified four quantifiable color variables on the body of the fish: the color of the body and the contrast of three distinct patterns (Fig. 2). Each pattern, when expressed, featured a whitish coloration, which contrasted with the darker purplish color of the body. The body color was measured at a specific location on the side of the fish, beneath the posterior end of the dorsal fin, where no pattern was ever apparent. The three patterns were measured as their contrast to their adjacent area on the body. We defined the three patterns as follows: (1) The side bars extend vertically downward from the base of the dorsal fin to about a third of the body depth. When fully displayed, five bars are visible, starting from the head and ending at the caudal peduncle (Fig. 2c). The bar located below the anterior part of the dorsal fin, above the gill opening, was always the most distinct bar. When the side bars pattern was dimmest and all other bars were invisible, the anterior bar almost always remained visible (Fig. 2b). (2) The head stripe is a long and narrow bar that extends from the lower jaw to the base of the dorsal fin (Fig. 2a). (3) The body patches are mottled blotches located on the lower side of the body and the caudal-peduncle (Fig. 2a).

Body color was sampled in a constant area on the body of the fish (Fig. 2a). The brightness calculation was performed by first extracting the RGB values for all pixels in the sampled area and then averaging them within each color channel. This procedure resulted in an RGB value of the mean intensities of the red, green, and blue color channels for the area ($\bar{I_{R}}$, $\bar{I_{G}}$, $\bar{I_{B}}$, respectively). The mean of $\bar{I_{R}}$, $\bar{I_{G}}$, $\bar{I_{B}}$ is referred to as the brightness of the area and ranges from 0 (darkest) to 1 (brightest) (Eq. 1).


$$\begin{array}{l} Body\;Color\;Brightness=\bar{\left(\bar{I_{R}},\;\bar{I_{G}},\;\bar{I_{B}}\right)} \end{array}$$ (1)

For the pattern contrast, the areas inside each pattern and the adjacent area outside it were sampled (Fig. 2a), and their brightness was calculated as explained above. The contrast between the areas was then calculated by subtracting the brightness of the adjacent area from the brightness inside the pattern, and dividing it by the brightness of the adjacent area (Eq. 2):


$$\begin{array}{l} Pattern\;Contrast=\frac{{\bar{\left(\bar{I_{R}},\bar{I_{G}},\bar{I_{B}}\right)}}_{inside}-{\bar{\left(\bar{I_{R}},\bar{I_{G}},\bar{I_{B}}\right)}}_{outside}}{{\bar{\left(\bar{I_{R}},\bar{I_{G}},\bar{I_{B}}\right)}}_{outside}} \end{array}$$ (2)

For the body patches pattern, contrast calculation was performed on the most distinctly visible patch in each image. For the side bars pattern, the contrast calculation was done on the two most anterior bars (always the most distinct bars), and then averaged. If a pattern was not displayed (too dim to identify such that the areas could not be marked), the contrast value was set to 0. If the location of a pattern was obstructed in an image, the value was set to NA.

### Error estimation

Some error in color reconstruction of images was expected due to inaccurate positioning of the color chart with respect to the location of the fish in the analyzed image. Such positioning inaccuracy can result from three types of deviations: distance (if the color chart was placed too close or too far from the fish), depth (if the color chart was placed deeper or shallower than the fish), and viewing angle (if the color chart was placed at a different angle than the fish, relative to the camera’s centerline, resulting in different exposure and light attenuation due to the position of the camera relative to the sun). Although it is difficult to precisely quantify these errors, we estimate that we placed the chart within a distance of ±0.25 m from the actual position of the fish (both along the distance and depth axes), and within ± 10 degrees angle. We quantified the potential error as a function of distance, depth, and viewing angle by placing the color chart at known distances and angles from a fish model (color-printed, laminated *V. louti* image). Error-quantification trials were conducted underwater at the study site, at a depth of 5 ± 0.5 m. In each trial, we first photographed the *V. louti* model alongside the color chart as a reference; and then moved the color chart closer or further; deeper or shallower, and at varying angles. Errors were expressed as percentages, calculated as the relative difference between the color value obtained from the reference image and the value obtained while calibrating the image with an inaccurately placed chart, divided by the value from the reference image. Our analysis was conducted for images differing in a single factor (distance, depth, and viewing angle) each time. From all of our measurements, we then calculated the expected error in color brightness and pattern contrast per meter (for distance and depth) or degree (for viewing angle).

Inaccurate placement of the color chart in terms of its distance from the camera had the greatest effect on the color standardization of the image, biasing the measured contrast by a rate of 34.1 ± 2.98% (mean ± SE) per meter (R^2^ = 0.88) and the brightness of the fish’s body color by 29.95 ± 2.84% per meter (R^2^ = 0.86). Inaccurate positioning of the chart with respect to the viewing angle biased the pattern contrast by 1.42 ± 0.36% per degree (R^2^ = 0.61) and the body color brightness by 1.01 ± 0.23% per degree (R^2^ = 0.65). Inaccurate placement of the color chart along the depth dimension had little effect on pattern contrast (1.91 ± 1.64% per meter, R^2^ = 0.12) or on body color brightness (8.59 ± 5.34% per meter, R^2^ = 0.21).

### Dataset composition and statistical analysis

Our data comprised 93 independent events that included 255 behavioral observations. Of the 255 total observations, it was possible to measure all four color pattern variables (body color brightness and the contrasts of body patches, side bars, and head stripe) in 184 cases, while in 66 observations the head stripe was not visible due to the imaging angle. In few observations, the body patches and side bars were obstructed by other fish or by the reef topography (4 and 1, respectively). Most observations (n = 213) featured *V. louti* alone, while the rest (n = 42) featured interspecific interactions.

We used MANOVA to test the effects of the behavioral variables on the three color-channels for the body color brightness and the contrasts of the pattern variables: body patches, side bars, and head stripe. The intensities in each of the three color-channels ($\bar{I_{R}}$, $\bar{I_{G}}$, $\bar{I_{B}}$) were the dependent variables, while the behavioral variables: swimming speed (4 levels), distance from the bottom (2 levels), and activity (6 levels) were the independent variables. These analyses indicated that the responses in the three color-channels were similar in direction and magnitude (Tables S1–S4 respectively). Therefore, we used linear mixed effects models to test the effects of the behavioral context on the averaged RGB values of the body color brightness (Eq. 1) and the contrasts of the of the body patches, side bars, and head stripe (Eq. 2). To account for potential confounding effects of samples taken from the same individuals, we included the ID of the fish as a random effect in the models.

To reduce the variance of potentially confounding effects between the behavioral variables, we repeated the linear mixed effect models on two subsets of the data: (1) While testing the effect of the activity on the color pattern variables, we excluded samples of fish swimming fast and positioned far from the bottom and ran the models again. (2) While testing the effects of swimming speed and distance from the bottom on the color pattern variables, we excluded all activities except for “none” from the dataset and run the models again. On both occasions, the results did not change the interpretation of the results (Tables S5 and S6). We therefore refer to the analyses on the full dataset in the results section. The significance of all LMER models (Tables 1–4, Table S5) was assessed using a likelihood ratio test between the full model and an intercept-only model that included only the random effect (Gałecki and Burzykowski 2013).

**Table 1.** LMER analysis of the behavioral effects on the brightness of the body color: BodyColor ~ Speed + Position + Activity + (1|Fish_ID).

Term	Sum Sq	Mean Sq	NumDF	DenDF	F value	Pr(> F)	Significance
Speed	0.035	0.012	3	206.29	2.47	0.063	.
Position	0.067	0.067	1	222.64	14.13	<0.001	***
Activity	0.023	0.005	5	180.61	0.96	0.442	

The model was significantly different than the intercept-only model: BodyColor ~ (1|Fish_ID); likelihood ratio test, χ^2^ = 31.06, *P* < 0.001.

Significance codes: ‘***’ 0.001, ‘.’ 0.1

**Table 2.** LMER analysis of the behavioral effects on the contrast of the body patches pattern: BodyPatches ~ Speed + Position + Activity + (1|Fish_ID).

Term	Sum Sq	Mean Sq	NumDF	DenDF	F value	Pr(> F)	Significance
Speed	1.889	0.63	3	222.92	57.97	<0.001	***
Position	0.006	0.006	1	231.87	0.54	0.462	
Activity	0.127	0.025	5	168.35	2.35	0.043	*

The model was significantly different than the intercept-only model: BodyPatches ~ (1|Fish_ID); likelihood ratio test, χ^2^ = 146.51, *P* < 0.001.

Significance codes: ‘***’ 0.001, ‘*’ 0.05

**Table 3.** LMER analysis of the behavioral effects on the contrast of the side bars pattern: SideBars ~ Speed + Position + Activity + (1|Fish_ID).

Term	Sum Sq	Mean Sq	NumDF	DenDF	F value	Pr(>F)	Significance
Speed	0.186	0.062	3	198.72	5.93	0.001	***
Position	0.086	0.086	1	217.1	8.28	0.004	**
Activity	0.031	0.006	5	196.68	0.6	0.702	

The model was significantly different than the intercept-only model: SideBars ~ (1|Fish_ID); likelihood ratio test, χ^2^ = 35.04, *P* < 0.001.

Significance codes: ‘***’ 0.001, ‘**’ 0.01

**Table 4.** LMER analysis of the behavioral effects on the contrast of the head stripe pattern: HeadStripe ~ Speed + Position + Activity + (1|Fish_ID).

Term	Sum Sq	Mean Sq	NumDF	DenDF	F value	Pr(>F)	Significance
Speed	0.054	0.018	3	172.94	2.9	0.037	*
Position	0.002	0.002	1	173.85	0.37	0.542	
Activity	1.034	0.207	5	139.66	33.16	<0.001	***

The model was significantly different than the intercept-only model: HeadStripe ~ (1|Fish_ID); likelihood ratio test, χ^2^ = 129.39, *P* < 0.001.

Significance codes: ‘***’ 0.001, ‘*’ 0.05

All statistical analyses were performed using R version 4.3.3 in RStudio version 2023.06.0.

### Rates of color change

We calculated the maximal rate of change in body color brightness and the contrast of body patches, side bars, and head stripe from image pairs taken less than 15 s apart (n = 71). For each pair we calculated the change in brightness or contrast (in %) and the rate of change (% per second; Fig. S2). The rates of change were calculated using the time durations between successive still images, extracted from the timestamp in the image metadata (providing a resolution of 1 s).

## Results

We observed rapid and extensive change in body color brightness (up to 83%) and pattern contrasts (up to 188%, 145%, and 100% for side bars, body patches, and head stripe, respectively) occurring within 15 s or less. The maximal rate of change was 12% s^-1^ for body color, 47% s^-1^ for side bars, 50% s^-1^ for body patches, and 25% s^-1^ for head stripe (Fig. S2). The magnitude and rate of change of body patches, side bars, and body color were all significantly different, with body patches being the highest and body color being the lowest in both (ANOVAs, *P* < 0.001 for both, Tables S7 and S8; Tukey post-hoc, *P* < 0.003 for all).

The correlations between the measured color pattern variables ranged from |0.04| to |0.44|, indicating an overall low dependence (Spearman correlation coefficient; Fig. S3).

### Behavioral context affects body color and patterns

**Fig. 4.**
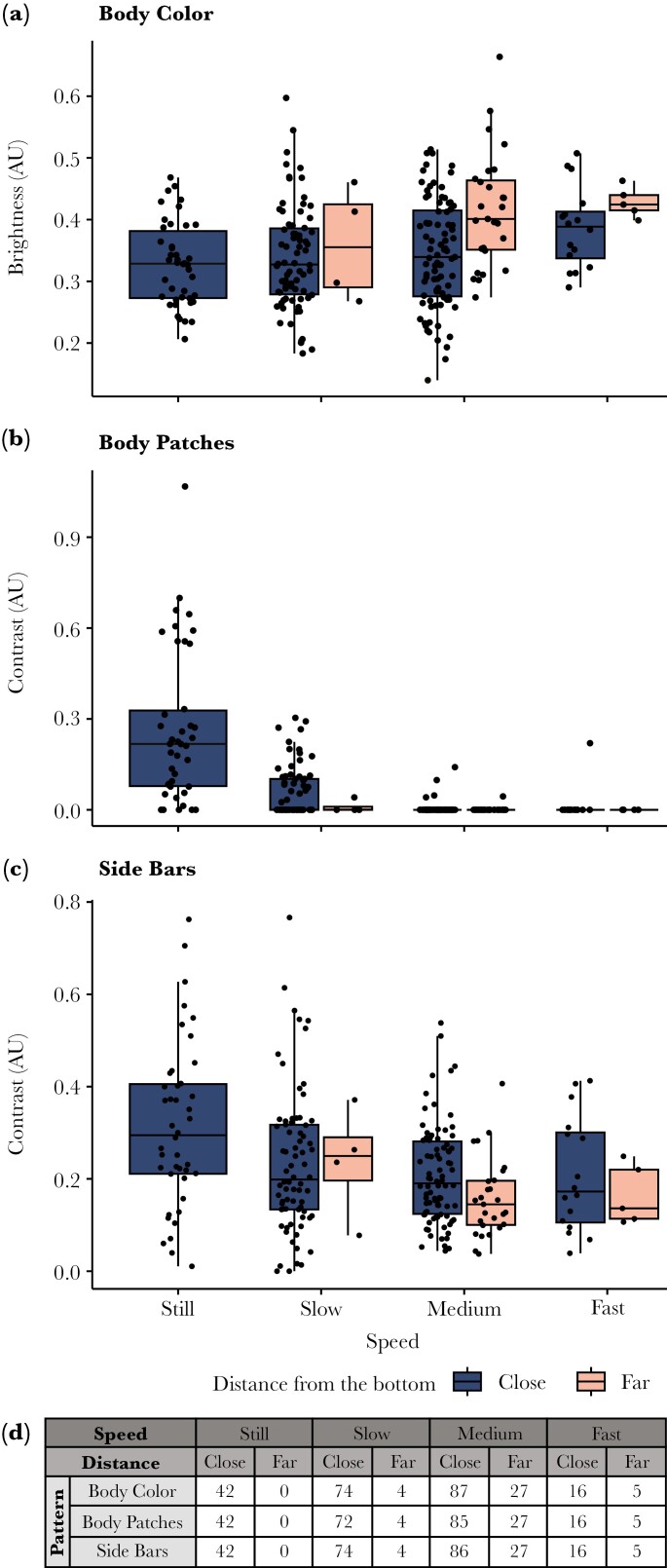
The effects of swimming speed and distance from the bottom on body color brightness (a), body patches contrast (b), and side bars contrast (c). *V. louti* brightened, lost its body patches, and decreased its side bars contrast with increasing swimming speeds and distance from the bottom. Sample sizes are listed in the table (d). Raw data points are plotted as black dots. The horizontal bold line marks the median. Lower and upper hinges correspond to the first and third quartiles. Whiskers represent values up to 1.5× inter-quartile range.

**Fig. 5.**
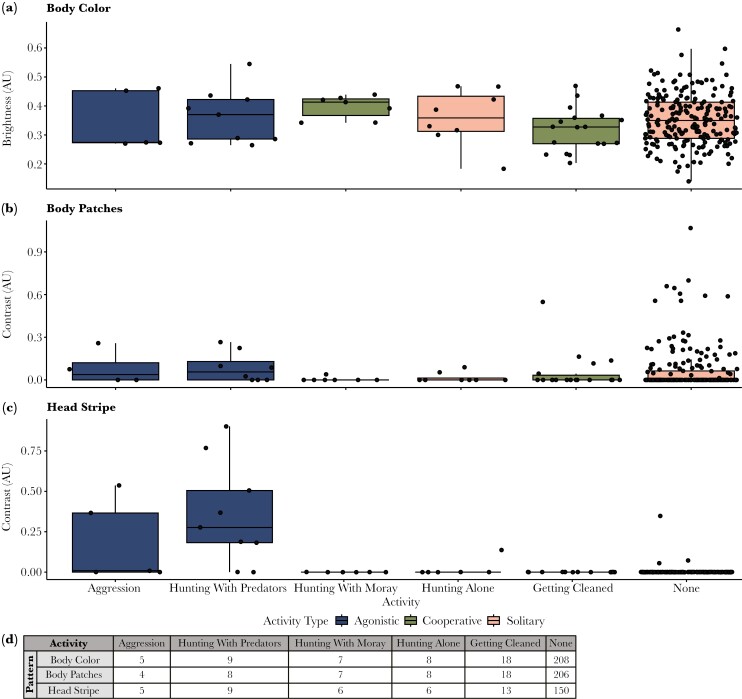
The effect of behavioral activity on the color patterns of *V. louti*. Shown are body color brightness (a) and the contrasts of body patches (b), and head stripe (c). Activities are colored by behavior type according to the legend. Sample sizes are listed in the table (d). Raw data points are plotted as black dots. The horizontal bold line marks the median. The lower and upper hinges correspond to the first and third quartiles. The whiskers represent values up to 1.5× inter-quartile range.

Distance from the bottom had a significant effect on body color brightness and swimming speed had a near-significant trend (LMER, *P* < 0.001 and *P* = 0.063 respectively; Table 1). *V. louti* displayed brighter body color with increasing swimming speeds and distance from the bottom (Fig. 4a). Body color was not affected by activity (LMER, *P* = 0.442, Table 1; Fig. 5a). Generally, the responses of the three color-channels that comprise the body color to the behavioral variables were similar (as indicated by their coefficients; MANOVA, Table S9). Therefore, we use the mean of the RGB channels as a measure of overall body-color brightness in our results.

The contrast of the body patches was significantly affected by speed and activity, but not by distance from the bottom (LMER, *P* < 0.001, *P* = 0.043, and *P* = 0.462 respectively; Table 2). The contrast was highest when the fish were still and close to the bottom (Fig. 4b; Video S1). However, a low contrast pattern was occasionally observed when the fish were close to the bottom and moving slowly (eg while coming to a stop or just starting to move). Body patches were not observed when the fish were hunting alone or with moray eels, whereas their median contrast was non-zero when engaged in aggressive interactions or hunting in association with other predators (Fig. 5b).

The contrast of the side bars decreased significantly with increasing swimming speed and distance from the bottom (Fig. 4c; LMER, *P* < 0.005 for both, Table 3), while the effect of the activity was not significant (LMER, *P* = 0.702, Table 3).

The contrast of the head stripe was significantly affected by activity and swimming speed (LMER, *P* < 0.001 and *P* = 0.0347 respectively, Table 4). The head stripe was displayed when *V. louti* was interacting with other predators: while engaged in aggressive encounters, and especially while hunting in close proximity to other predators (Fig. 5c; *P* < 0.01 for aggressive encounters versus all other activities, and *P* < 0.005 for hunting in close proximity with other predators versus all other activities, Tukey post-hoc, Table 5).

**Table 5.** Summary of Tukey post-hoc analyses of the four models in Tables 1–4. Significant values are highlighted. Full post-hoc tables are available in the supplementary information (Table S6).

Term	Pairwise	Body Color	Body Patches	SIde Bars	Head Stripe
Speed	Fast—Medium	0.302	0.997	0.998	0.887
Speed	Fast—Slow	0.095	0.333	0.798	0.831
Speed	Fast—Still	0.059	**<0.001**	**0.016**	0.936
Speed	Medium—Slow	0.711	**0.017**	0.646	**0.033**
Speed	Medium—Still	0.505	**<0.001**	**<0.001**	0.194
Speed	Slow—Still	0.964	**<0.001**	**0.019**	0.984
Position	Close—Far	**<0.001**	0.466	**0.005**	0.546
Activity	aggression—cleaning	0.84	1	1	**0.001**
Activity	aggression—hunting alone	1	0.933	1	**0.005**
Activity	aggression—hunting with moray	0.998	0.623	0.98	**0.009**
Activity	aggression—hunting with predators	1	0.961	1	**0.004**
Activity	aggression—none	0.966	1	0.999	**<0.001**
Activity	cleaning—hunting alone	0.712	0.697	0.999	1
Activity	cleaning—hunting with moray	0.64	0.269	0.991	1
Activity	cleaning—hunting with predators	0.793	0.889	1	**<0.001**
Activity	cleaning—none	0.938	1	0.872	1
Activity	hunting alone—hunting with moray	0.999	0.971	0.97	1
Activity	hunting alone—hunting with predators	1	0.205	1	**<0.001**
Activity	hunting alone—none	0.899	0.476	0.999	1
Activity	hunting with moray—hunting with predators	0.996	0.087	0.99	**<0.001**
Activity	hunting with moray—none	0.82	0.138	0.862	1
Activity	hunting with predators—none	0.951	0.856	0.985	**<0.001**

Interestingly, *V. louti* expressed different patterns while hunting with moray eels than while hunting in association with other predatory species, albeit similar to those expressed when hunting alone (Fig. 5b, c). Specifically, the contrast of the head stripe when hunting with a moray eel was significantly lower compared to when hunting in association with other predators, but not significantly different than when hunting alone or being cleaned (*P* < 0.001, *P* > 0.999, and *P* > 0.999 respectively, Tukey post-hoc, Table 5).

### Interspecific hunting cooperation between *Variola louti* and two species of moray eels

The interactions of *V. louti* with moray eels matched published descriptions of cooperative hunting between other species of groupers and moray eels (Bshary et al. 2006; Vail et al. 2013). We obtained recordings documenting six occasions of a head-shake signaling gesture by *Variola louti* toward two species of moray eels (*Gymnothorax griseus* and *Gymnothorax nudivomer*), followed by the morays joining the grouper in a cooperative hunt (Fig. 1, Video S2).

## Discussion

Dynamic color changes in Serranids are mostly known in the context of courtship behaviors (Clark 1959; Barlow 1975; Colin 1992; Gilmore and Jones 1992; Sadovy and Eklund 1999; Erisman and Allen 2005; Archer et al. 2012) and camouflage (Townshead 1929; Watson et al. 2014). Evidence of rapid color changes in interspecies interactions in fish are generally rare, especially those related to agonistic signaling. Here we have shown that *Variola louti* assumed different color appearances by rapidly changing distinct patterns on their body in accordance with their behavior, most notably in aggressive or hunting interactions with other predatory species, and according to swimming speed and proximity to the substrate (Fig. 1; Video S1). *V. louti* dynamically displayed a range of body color brightness. Similarly, the contrast of the side bars, body patches, and head stripe varied from completely invisible to intensely displayed (Fig. 2). These four color variables were largely expressed independently of each other, and could thus form numerous combinations of visual displays (Fig. S3). We have also shown that *V. louti* was able to change its colors rapidly, with substantial color transformations taking place in mere seconds (Fig. 2, Fig. S2).

*V. louti* changed their body color brightness and the contrast of the body patches and side bars according to their swimming speed and proximity to the substrate (Fig. 4). They also intensely expressed the head stripe during competitive or aggressive interactions with predatory species, but not when hunting alone or with moray eels (Fig. 5c). These findings are consistent with our discovery of cooperative hunting between *V. louti* and moray eels (Fig. 1; Video S2).

Below we discuss the color patterns associated with three behavior types: agonistic interactions, solitary swimming and hunting, and cooperative hunting. We then discuss the color patterns that changed in correlation with swimming speed and distance from the bottom, suggesting the use of camouflage by disruptive coloration and background matching.

### Behavioral context of dynamic head stripe expression suggests interspecific agonistic signaling

The head stripe was displayed almost exclusively during interspecific hunting associations and aggressive interactions with other predatory species (Fig. 5c), with the exception of cooperative hunting with moray eels (see the next subsection). The contrast of the body patches was altered similarly, although less intensely (Fig. 5b). Interspecies hunting associations are not a rare occurrence in coral reefs, and involve individuals from multiple species that hunt in close proximity and potentially compete for prey (Diamant and Shpigel 1985; Lukoschek and McCormick 2000). The bright white head stripe of *V. louti*, stretching along the dorsal part of the head, might be expressed as an agonistic signal when the fish are disturbed or threatened. Agonistic signals via color displays in fish are mostly known to be directed at conspecifics, in territorial (Korzan and Fernald 2007) or courtship contexts (Kodric-Brown 1998). However, to the best of our knowledge there is no evidence linking dynamic color displays to agonistic signaling towards interspecifics. *V. louti* expressed the head stripe toward other groupers (*Cephalopholis miniata*, *Epinephelus tauvina*, *Epinephelus fasciatus*, *Aethaloperca rogaa*), large gold-saddle goatfish (*Parupeneus cyclostomus*), devil firefish (*Pterois miles*) and big blue octopus (*Octopus cyanea*) (Video S1). To the best of our knowledge, this is the first evidence of context-dependent color displays by a Serranid that may serve for interspecific signaling, although the question remains as to whether and how the recipients perceive and respond to these head stripe displays.

Interestingly, we documented four occasions of octopuses (*Octopus cyanea*) displaying a long white stripe across their head in competitive interspecies hunting associations with other predators (Fig. 1a, Video S3), similarly to *V. louti*’s display of the head stripe, perhaps also displayed as an agonistic signal. We found further evidence of the head stripe pattern for both *V. louti* and *O. cyanea* in a supplementary video from Sampaio et al. (2020), in which the authors describe an agonistic “punching” gesture by octopuses towards fishes in interspecific hunting events. The video features eight punching events by octopuses during interspecific hunting associations, two of which show octopuses displaying a head stripe, and four show *V. louti* displaying a head stripe. More research is required to understand the expression of the head stripe as a potential convergent signal by phylogenetically distant species. Specifically, the reactions of the recipients to the head stripe display should be studied to better understand the functionality of the signal.

### Color patterns during interactions with moray eels align with findings of cooperative hunting behavior

In interspecific cooperative interactions, participants can benefit from the diverse abilities of the different species involved (Axelrod and Hamilton 1981; Minta et al. 1992; Lukoschek and McCormick 2000; Bshary et al. 2006). Complementary hunting skills, arising from differences in morphology and hunting techniques, could improve efficiency in the form of higher success rate, a wider variety of catchable prey, and less energy and time spent by each individual hunter (Minta et al. 1992).

Communicative cooperative hunting between groupers and moray eels has been previously reported between the roving coral grouper (*Plectropomus pessuliferus*) and the giant moray (*Gymnothorax javanicus*) in the Red Sea (Bshary et al. 2006; Vail et al. 2013). Typically, the grouper initiated the cooperation by shaking its head as an invitation signal to the moray. The moray then left its crevice and the pair started swimming together in search of prey. The grouper occasionally directed the moray to the prey’s location by performing head shakes, while pointing down at the crevice where the prey was hiding (Bshary et al. 2006; Vail et al. 2013). This joint effort allowed the pair to increase their success rate by flanking their prey, as the moray crawled into the crevice where the prey was hiding, while the grouper assumed a position from above in case the prey escaped outwards (Bshary et al. 2006; Vail et al. 2013).

Here, we documented for the first time communicative cooperative hunting of *V. louti* with two previously unreported cooperator species: *Gymnothorax griseus* and *Gymnothorax nudivomer* (Fig. 1, Video S2). Bshary et al. (2006) had observed *V. louti* head-shaking once towards *G. javanicus* and once towards *G. griseus*, but they did not report cooperative hunting. The interactions we observed included the same characteristics described in the previous reports of communicative hunting cooperation between other species of groupers and moray eels, including head-shake signaling by the grouper, followed by a joint hunt by the pair. In line with these findings, *V. louti* did not express the potentially agonistic head stripe when hunting in cooperation with moray eels nor when hunting alone, as opposed to when hunting in association with other predators (Fig. 5c). This may be attributed to *V. louti* considering morays as allies rather than competitors, further supporting our findings of cooperative hunting and the head stripe as an agonistic signal.

### Motion and benthic proximity appear to trigger camouflage colorations

When *V. louti* was stationary and positioned close to the bottom, its body color appeared dark with bright mottled patches and vertical side bars (Fig. 4), potentially camouflaging via disruptive coloration in the visually complex and non-uniform environment of the reef. On the contrary, as *V. louti* was swimming faster and farther away from the bottom, it lost the body patches, dimmed the side bars, and brightened the body color (Fig. 4), likely matching its appearance to the uniform bright background of the open water (Fig. S4). Transitions between swimming speeds and distances from the bottom were consistently accompanied by color and pattern changes (Fig. 2; Video S1).

Disruptive coloration and background matching are two effective mechanisms for achieving camouflage (Thayer 1918; Cott 1940; Cuthill et al. 2005; Troscianko et al. 2021). In background matching the animal’s colors closely resemble a sample of the background in color and geometry (Cott 1940), while in disruptive coloration, contrasting colors on the animal’s body break up its outline, making it more difficult to identify (Thayer 1918; Cott 1940; Stevens and Merilaita 2008). Disruptive coloration is a widespread camouflage mechanism across animals (Stevens et al. 2006) and is thought to be at least as effective as background matching (Cuthill et al. 2005; Schaefer and Stobbe 2006; Stevens et al. 2006). In visually complex environments, disruptive coloration is less sensitive to the object’s movement than background matching. Contrary, for a moving object on a uniform background, disruptive coloration becomes less useful, making background matching preferable for camouflage, as is the case for fish in the featureless background of open water (Marshall and Johnsen 2011). Although we did not quantify the color properties of the background, and therefore cannot quantitatively determine that background matching was being used, we suggest that *V. louti* is potentially alternating between disruptive coloration when stationary near the seabed, and background matching when swimming in more open water.

## Supplementary Material

araf005_suppl_Supplementary_Materials

## Data Availability

Analyses reported in this article can be reproduced using the data provided by Marom et al. 2025, at Dryad: https://doi.org/10.5061/dryad.n5tb2rc5t.
