# Supplementary material for: Dynamic color change in the grouper Variola louti during interspecific interactions and swimming
Source: Behav Ecol. 2025 Jan 20;36(2):araf005. doi: 10.1093/beheco/araf005 (PMC11851106; doi:10.1093/beheco/araf005)
Supplement: araf005_suppl_Supplementary_Materials [file araf005_suppl_supplementary_materials.zip › Variola paper 11012025 - Supplementary/Variola paper 11012025 - Supplementary.pdf]

Dynamic color change in the grouper *Variola louti* during interspecific interactions and swimming

Authors

Sagi Marom[1,4], Moshe Kiflawi[2,4], Derya Akkaynak[3,4], Roi Holzman[1,4,5]

1. School of Zoology, Faculty of Life Sciences, Tel Aviv University, Tel Aviv 69978, Israel.

2. Department of Life Sciences, Ben Gurion University, Beer Sheva 8410501, Israel.

3. Hatter Department of Marine Technologies, Leon H. Charney School of Marine Sciences, University of Haifa, Haifa, 3103301, Israel.

4. The Inter-University Institute for Marine Sciences, PO Box 469, Eilat 88103, Israel.

5. Steinhardt Museum of Natural History, Tel Aviv University, Tel Aviv, Israel.

Corresponding author

Sagi Marom. Email address: sagimarom@mail.tau.ac.il

Supplementary Information

- Videos:
    - S1 - *Variola louti* color changes.
      Original video:
      https://drive.google.com/file/d/1wh4WbawKOmJsMWH-WplZz9e4XfB4UHke/view?usp=sharing
      YouTube:
      https://youtu.be/XLYwR9Di11g
    - S2 - Hunting cooperation between *Variola louti* to *Gymnothorax griseus* and *Gymnothorax nudivomer*.
      Original video:
      https://drive.google.com/file/d/18O5Y3pxbEQaJgdJV2WY3kStc36QnsGG6/view?usp=sharing
      YouTube:
      https://youtu.be/8tDs3doNSs0
    - S3 - Head stripe display by *Octopus cyanea*.
      Original video:
      https://drive.google.com/file/d/1D2yuFcZZjSN9cLb4uCrMhBk9lT_RFR1R/view?usp=sharing
      YouTube:
      https://youtu.be/rAo7Gg0U6Xw
- Supplementary figures.
- Supplementary tables.

Supplementary figures

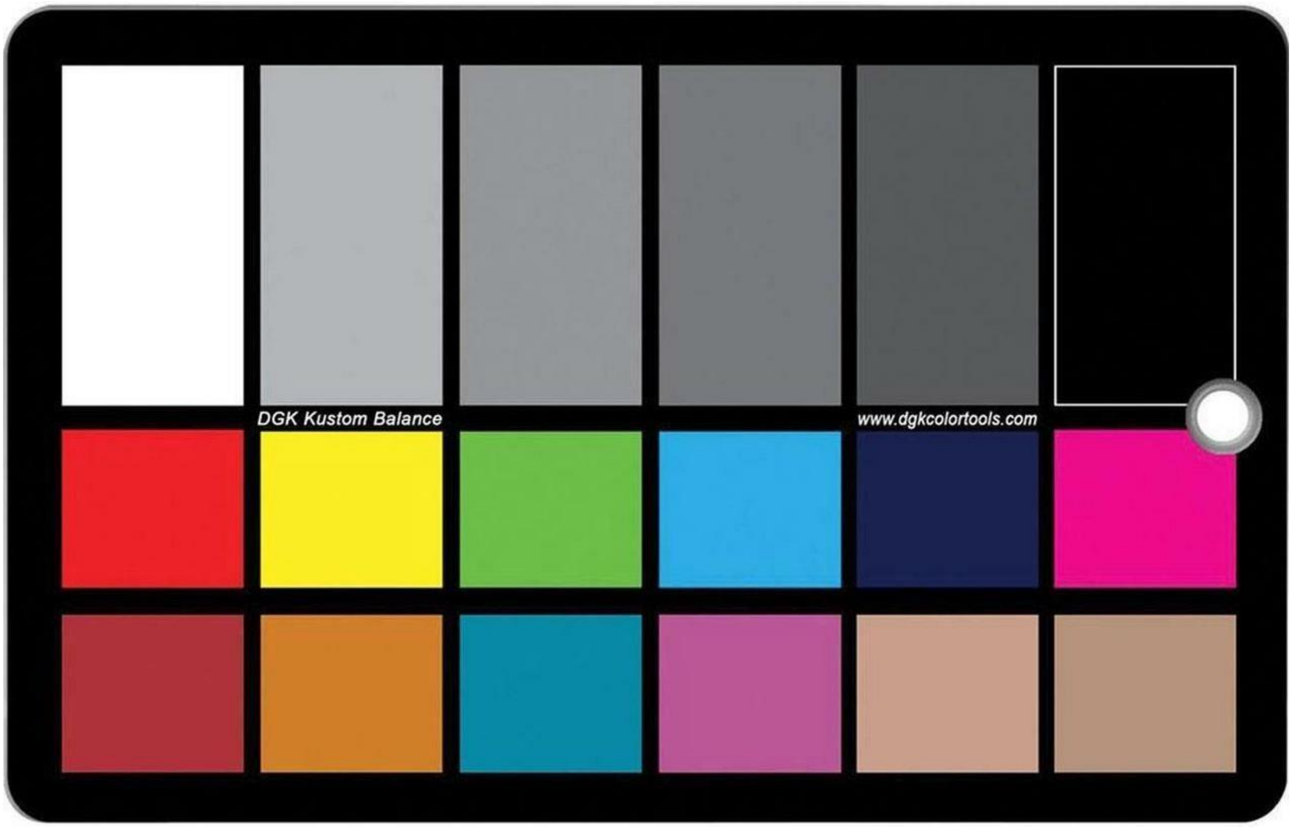

Figure S1: The waterproof color chart we used - DGK Color Tools WDKK. For the color standardization we used the third pallet from the left in the upper row (18% gray).

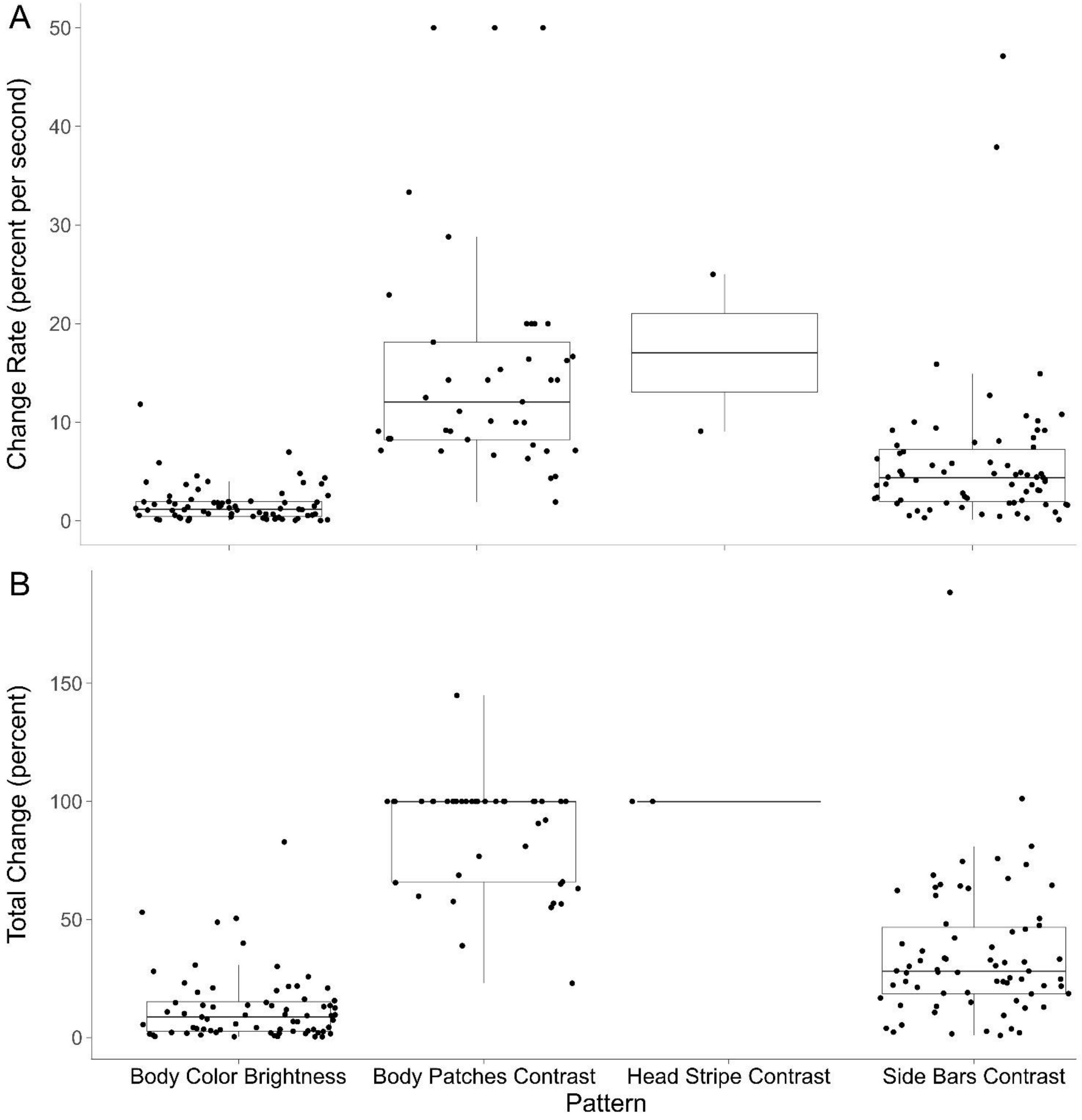

C

Pattern	Body Color	Body Patches	Head Stripe	Side Bars
Change Rate	71	41	2	71
Total Change	71	41	2	71

Figure S2: Pattern change events of up to 15 seconds long (n=71). Shown are (A) the rate of change (%s^{-1}) and (B) the change % per event for each pattern. Sample sizes are listed in the table (C). Raw data points of the events are plotted as black dots. The horizontal bold line marks the median. Lower and upper hinges correspond to the first and third quartiles. Whiskers represent values up to 1.5 × inter-quartile range.

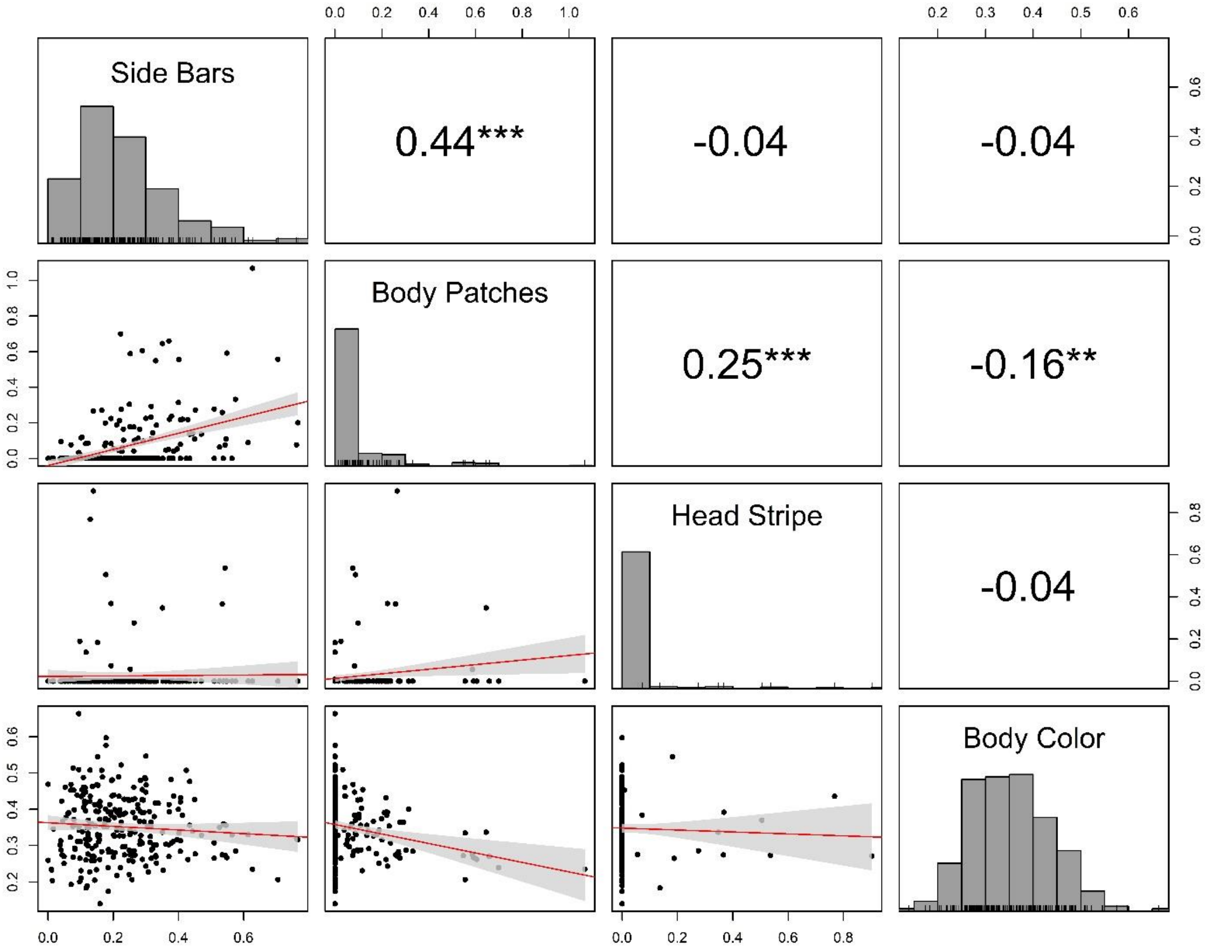

Figure S3: Correlation plot between the four measured color variables. Gray bars are histogram plots of each variable. The diagonal contains the univariate distribution of each variable. The cells below the diagonal are bivariate plots of each two variables, and the numbers in the cells above the diagonal are Spearman's correlation coefficients. Significance Codes: '***' 0.001, '**' 0.01

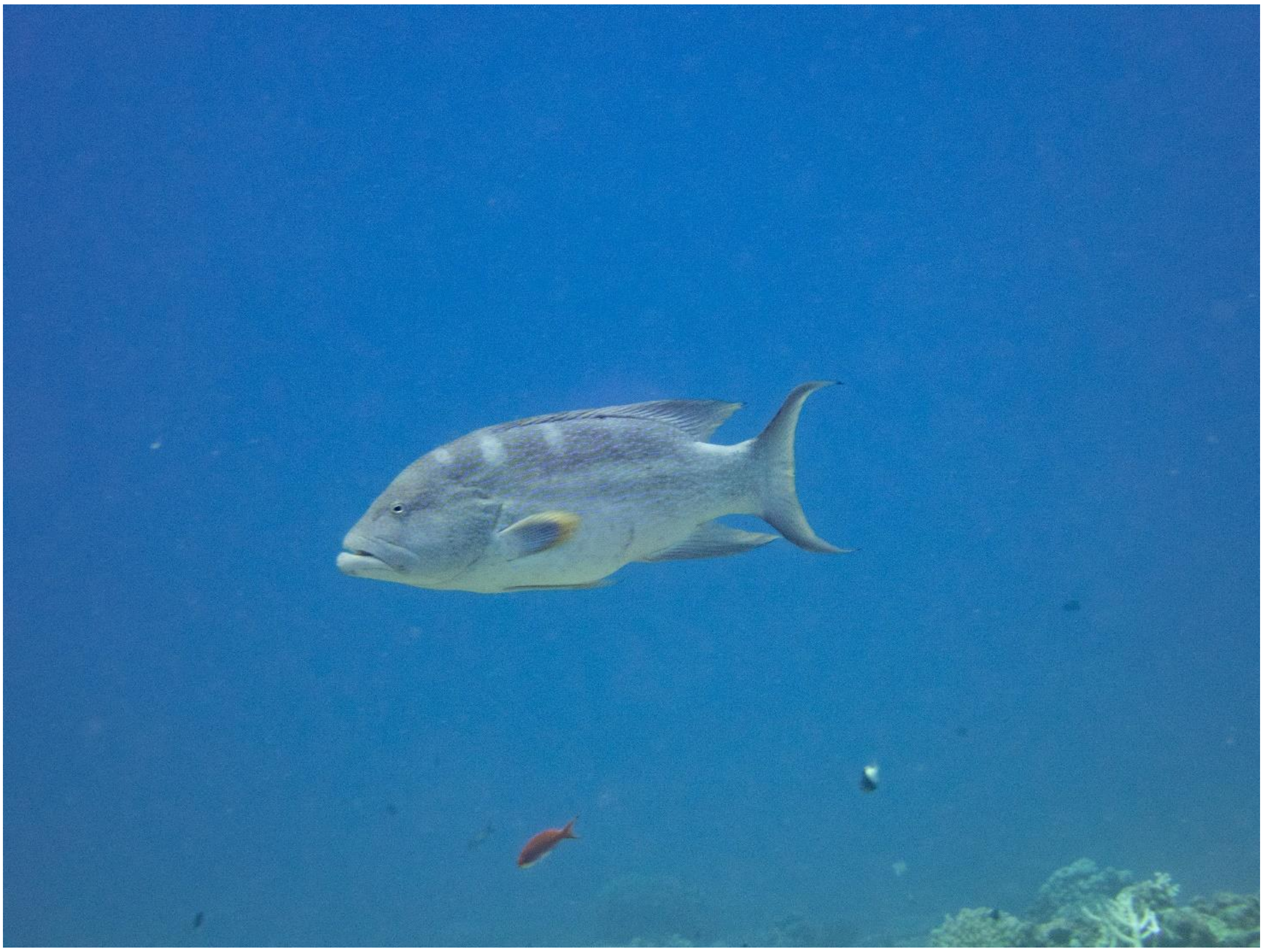

Figure S4: *Variola louti* swimming far from the bottom, expressing a bright body color with no body patches and faint side bars, potentially background matching to the uniform bright background of the open water.

Supplementary tables

Table S1: The effect of behavioral variables on the body color, analyzed separately for each color channel (RGB). Data are estimated coefficients from MANOVA analysis.

**Term**	**BodyColor_R**	**BodyColor_G**	**BodyColor_B**
Speed: Fast	0.460865643	0.33841843	0.35764712
Speed: Medium	-0.023870875	-0.038284	-0.0358788
Speed: Slow	-0.023461641	-0.0548395	-0.055574
Speed: Still	-0.024599523	-0.0657208	-0.0735663
Position: Close	0.460865643	0.33841843	0.35764712
Position: Far	0.03356296	0.0756431	0.07563353
Activity: Aggression	0.460865643	0.33841843	0.35764712
Activity: Cleaning	-0.033710547	-0.0314269	-0.0321944
Activity: Hunting alone	0.005714762	0.02477152	0.02799311
Activity: Hunting with moray	0.018523975	0.06310742	0.06912798
Activity: Hunting with predators	-0.00223168	0.02792831	0.03122673
Activity: None	-0.013263604	-0.0035286	-0.0030927

Table S2: The effect of behavioral variables on the contrast of the body patches pattern, analyzed separately for each color channel (RGB). Data are estimated coefficients from MANOVA analysis.

**Term**	**BodyPatches_R**	**BodyPatches_G**	**BodyPatches_B**
Speed: Fast	0.010644434	0.032081207	0.005518763
Speed: Medium	-0.003293002	-0.018009646	-0.017281265
Speed: Slow	0.019563856	0.069577678	0.052929605
Speed: Still	0.096050402	0.445320669	0.373840398
Position: Close	0.010644434	0.032081207	0.005518763
Position: Far	-0.005163658	-0.020419256	-0.019019355
Activity: Aggression	0.010644434	0.032081207	0.005518763
Activity: Cleaning	-0.006495207	-0.009018054	0.018137921
Activity: Hunting alone	-0.029543422	-0.12010532	-0.078269521
Activity: Hunting with moray	-0.05069976	-0.217144411	-0.163617641
Activity: Hunting with predators	0.015000323	0.070339941	0.082833943
Activity: None	-0.003948689	-0.001591437	0.022832465

Table S3: The effect of behavioral variables on the contrast of the side bars pattern, analyzed separately for each color channel (RGB). Data are estimated coefficients from MANOVA analysis.

**Term**	**SideBars_R**	**SideBars_G**	**SideBars_B**
Speed: Fast	0.107585491	0.3429849	0.30270506
Speed: Medium	0.001824493	0.019522753	0.004077093
Speed: Slow	0.010917138	0.068291156	0.056290497
Speed: Still	0.031700649	0.167168107	0.174576488
Position: Close	0.107585491	0.3429849	0.30270506
Position: Far	-0.026971812	-0.070563536	-0.045777359
Activity: Aggression	0.107585491	0.3429849	0.30270506
Activity: Cleaning	-0.041519878	-0.136824715	-0.106245438
Activity: Hunting alone	-0.008902646	-0.062169621	-0.108949686
Activity: Hunting with moray	-0.04370955	-0.204728446	-0.203607189
Activity: Hunting with predators	-0.038279565	-0.135126413	-0.108720252
Activity: None	-0.009340473	-0.042784433	-0.038140833

Table S4: The effect of behavioral variables on the contrast of the head stripe pattern, analyzed separately for each color channel (RGB). Data are estimated coefficients from MANOVA analysis.

**Term**	**HeadStripe_R**	**HeadStripe_G**	**HeadStripe_B**
Speed: Fast	0.078686086	0.276507853	0.24602843
Speed: Medium	-0.006648679	-0.023922033	-0.019999735
Speed: Slow	0.011124781	0.039223793	0.031149375
Speed: Still	0.00666519	0.029103295	0.028717537
Position: Close	0.078686086	0.276507853	0.24602843
Position: Far	0.005320647	0.019038716	0.015940869
Activity: Aggression	0.078686086	0.276507853	0.24602843
Activity: Cleaning	-0.080649823	-0.283194564	-0.250862198
Activity: Hunting alone	-0.079536278	-0.270556626	-0.24619503
Activity: Hunting with moray	-0.08054572	-0.284772239	-0.254125927
Activity: Hunting with predators	0.08006398	0.249977317	0.158426374
Activity: None	-0.079968114	-0.280948923	-0.249639804

Table S5: LMER analyses of the behavioral effects on the color pattern variables, performed on two subsets of the original dataset.

Significance codes: '***'0.001, '**'0.01, '*'0.05, '.' 0.1

Dataset	Model	Term	Sum Sq	Mean Sq	Num DF	Den DF	F value	Pr(>F)	Sig.
Only "none" activity	BodyColor ~ Speed+Position+(1lFishID)	Speed	0.041	0.014	3	151.3	3.48	0.018	*
Only "none" activity	BodyColor ~ Speed+Position+(1lFishID)	Position	0.049	0.049	1	171.12	12.46	0.001	***
Only "none" activity	BodyPatches ~ Speed+Position+(1lFishID)	Speed	1.855	0.618	3	185.14	56.59	<0.001	***
Only "none" activity	BodyPatches ~ Speed+Position+(1lFishID)	Position	0.003	0.003	1	193.32	0.32	0.572	
Only "none" activity	SideBars ~ Speed+Position+(1lFishID)	Speed	0.234	0.078	3	161.48	7.96	<0.001	***
Only "none" activity	SideBars ~ Speed+Position+(1lFishID)	Position	0.085	0.085	1	182.08	8.66	0.004	**
Only "none" activity	HeadStripe ~ Speed+Position+(1lFishID)	Speed	0.003	0.001	3	138.79	1.26	0.292	
Only "none" activity	HeadStripe ~ Speed+Position+(1lFishID)	Position	<0.001	<0.001	1	138.37	<0.01	0.948	
No "fast" speed, No "far" Position	BodyColor ~ Speed+Activity+(1lFishID)	Speed	0.006	0.003	2	158.41	0.65	0.521	
No "fast" speed, No "far" Position	BodyColor ~ Speed+Activity+(1lFishID)	Activity	0.021	0.004	5	131.92	0.99	0.429	
No "fast" speed, No "far" Position	BodyPatches ~ Speed+Activity+(1lFishID)	Speed	1.749	0.875	2	171.91	68.48	<0.001	***
No "fast" speed, No "far" Position	BodyPatches ~ Speed+Activity+(1lFishID)	Activity	0.131	0.026	5	122.12	2.04	0.077	.
No "fast" speed, No "far" Position	SideBars ~ Speed+Activity+(1lFishID)	Speed	0.187	0.093	2	161.74	7.86	0.001	***
No "fast" speed, No "far" Position	SideBars ~ Speed+Activity+(1lFishID)	Activity	0.04	0.008	5	141.52	0.67	0.65	
No "fast" speed, No "far" Position	HeadStripe ~ Speed+Activity+(1lFishID)	Speed	0.057	0.029	2	145.37	3.88	0.023	*
No "fast" speed, No "far" Position	HeadStripe ~ Speed+Activity+(1lFishID)	Activity	1.026	0.205	5	111.98	27.89	<0.001	***

Table S6: Tukey post-hoc analyses results of the LMER analyses in tables 1-4 and table S5.

Significance codes: '***'0.001, '**'0.01, '*'0.05, '.' 0.1

Dataset	Model	Term	pairwise	estimate	SE	df	t.ratio	p.value	Sig.
Full data	BodyColor ~ Speed+Position+Activity+(1|FishID)	Speed	Fast - Medium	0.032	0.018	211.54	1.75	0.302	
Full data	BodyColor ~ Speed+Position+Activity+(1|FishID)	Speed	Fast - Slow	0.044	0.019	209.36	2.33	0.095	.
Full data	BodyColor ~ Speed+Position+Activity+(1|FishID)	Speed	Fast - Still	0.052	0.02	206.1	2.52	0.059	.
Full data	BodyColor ~ Speed+Position+Activity+(1|FishID)	Speed	Medium - Slow	0.013	0.012	218.62	1.07	0.711	
Full data	BodyColor ~ Speed+Position+Activity+(1|FishID)	Speed	Medium - Still	0.02	0.014	211.15	1.39	0.505	
Full data	BodyColor ~ Speed+Position+Activity+(1|FishID)	Speed	Slow - Still	0.007	0.015	206.5	0.48	0.964	
Full data	BodyColor ~ Speed+Position+Activity+(1|FishID)	Position	Close - Far	-0.055	0.015	225.24	-3.74	<0.001	***
Full data	BodyColor ~ Speed+Position+Activity+(1|FishID)	Activity	aggression - cleaning	0.046	0.038	201.78	1.19	0.84	
Full data	BodyColor ~ Speed+Position+Activity+(1|FishID)	Activity	aggression - hunting alone	-0.003	0.044	198.57	-0.08	1	
Full data	BodyColor ~ Speed+Position+Activity+(1|FishID)	Activity	aggression - hunting with moray	-0.023	0.053	184.3	-0.44	0.998	
Full data	BodyColor ~ Speed+Position+Activity+(1|FishID)	Activity	aggression - hunting with predators	0.002	0.043	208.29	0.05	1	
Full data	BodyColor ~ Speed+Position+Activity+(1|FishID)	Activity	aggression - none	0.027	0.034	185.42	0.81	0.966	
Full data	BodyColor ~ Speed+Position+Activity+(1|FishID)	Activity	cleaning - hunting alone	-0.049	0.034	230.86	-1.43	0.712	
Full data	BodyColor ~ Speed+Position+Activity+(1|FishID)	Activity	cleaning - hunting with moray	-0.069	0.045	117.49	-1.54	0.64	
Full data	BodyColor ~ Speed+Position+Activity+(1|FishID)	Activity	cleaning - hunting with predators	-0.043	0.034	237.74	-1.29	0.793	
Full data	BodyColor ~ Speed+Position+Activity+(1|FishID)	Activity	cleaning - none	-0.018	0.02	235.5	-0.93	0.938	
Full data	BodyColor ~ Speed+Position+Activity+(1|FishID)	Activity	hunting alone - hunting with moray	-0.02	0.05	151.56	-0.4	0.999	
Full data	BodyColor ~ Speed+Position+Activity+(1|FishID)	Activity	hunting alone - hunting with predators	0.006	0.036	206.86	0.16	1	
Full data	BodyColor ~ Speed+Position+Activity+(1|FishID)	Activity	hunting alone - none	0.031	0.029	219.93	1.05	0.899	
Full data	BodyColor ~ Speed+Position+Activity+(1|FishID)	Activity	hunting with moray - hunting with predators	0.025	0.049	140.25	0.52	0.996	
Full data	BodyColor ~	Activity	hunting with moray -	0.05	0.041	94.13	1.23	0.82	

	Speed+Position+Activity+(1|FishID)		none						
Full data	BodyColor ~ Speed+Position+Activity+(1|FishID)	Activity	hunting with predators - none	0.025	0.028	235.95	0.88	0.951	
Full data	BodyPatches ~ Speed+Position+Activity+(1|FishID)	Speed	Fast - Medium	0.005	0.026	223.29	0.21	0.997	
Full data	BodyPatches ~ Speed+Position+Activity+(1|FishID)	Speed	Fast - Slow	-0.047	0.028	222.3	-1.69	0.333	
Full data	BodyPatches ~ Speed+Position+Activity+(1|FishID)	Speed	Fast - Still	-0.261	0.03	222.08	-8.74	<0.001	***
Full data	BodyPatches ~ Speed+Position+Activity+(1|FishID)	Speed	Medium - Slow	-0.053	0.018	226.22	-2.98	0.017	*
Full data	BodyPatches ~ Speed+Position+Activity+(1|FishID)	Speed	Medium - Still	-0.266	0.021	223.42	-12.76	<0.001	***
Full data	BodyPatches ~ Speed+Position+Activity+(1|FishID)	Speed	Slow - Still	-0.214	0.022	219.69	-9.81	<0.001	***
Full data	BodyPatches ~ Speed+Position+Activity+(1|FishID)	Position	Close - Far	0.016	0.021	231.9	0.73	0.466	
Full data	BodyPatches ~ Speed+Position+Activity+(1|FishID)	Activity	aggression - cleaning	-0.005	0.062	225.52	-0.08	1	
Full data	BodyPatches ~ Speed+Position+Activity+(1|FishID)	Activity	aggression - hunting alone	0.067	0.07	231.54	0.95	0.933	
Full data	BodyPatches ~ Speed+Position+Activity+(1|FishID)	Activity	aggression - hunting with moray	0.117	0.075	175.88	1.56	0.623	
Full data	BodyPatches ~ Speed+Position+Activity+(1|FishID)	Activity	aggression - hunting with predators	-0.059	0.07	233.99	-0.83	0.961	
Full data	BodyPatches ~ Speed+Position+Activity+(1|FishID)	Activity	aggression - none	-0.009	0.056	222.21	-0.16	1	
Full data	BodyPatches ~ Speed+Position+Activity+(1|FishID)	Activity	cleaning - hunting alone	0.071	0.049	233.03	1.45	0.697	
Full data	BodyPatches ~ Speed+Position+Activity+(1|FishID)	Activity	cleaning - hunting with moray	0.122	0.057	97.37	2.15	0.269	
Full data	BodyPatches ~ Speed+Position+Activity+(1|FishID)	Activity	cleaning - hunting with predators	-0.054	0.05	218.79	-1.08	0.889	
Full data	BodyPatches ~ Speed+Position+Activity+(1|FishID)	Activity	cleaning - none	-0.004	0.028	233.88	-0.14	1	
Full data	BodyPatches ~ Speed+Position+Activity+(1|FishID)	Activity	hunting alone - hunting with moray	0.05	0.065	128.6	0.78	0.971	
Full data	BodyPatches ~ Speed+Position+Activity+(1|FishID)	Activity	hunting alone - hunting with predators	-0.125	0.055	216.19	-2.28	0.205	
Full data	BodyPatches ~ Speed+Position+Activity+(1|FishID)	Activity	hunting alone - none	-0.075	0.042	233.75	-1.79	0.476	
Full data	BodyPatches ~ Speed+Position+Activity+(1|FishID)	Activity	hunting with moray - hunting with predators	-0.176	0.065	115.83	-2.68	0.087	.
Full data	BodyPatches ~ Speed+Position+Activity+(1|FishID)	Activity	hunting with moray - none	-0.126	0.05	72.07	-2.5	0.138	

Full data	BodyPatches ~ Speed+Position+Activity+(1|FishID)	Activity	hunting with predators - none	0.05	0.043	220.3	1.16	0.856	
Full data	SideBars ~ Speed+Position+Activity+(1|FishID)	Speed	Fast - Medium	-0.005	0.027	199.15	-0.18	0.998	
Full data	SideBars ~ Speed+Position+Activity+(1|FishID)	Speed	Fast - Slow	-0.026	0.029	197.32	-0.91	0.798	
Full data	SideBars ~ Speed+Position+Activity+(1|FishID)	Speed	Fast - Still	-0.093	0.031	192.83	-3	0.016	*
Full data	SideBars ~ Speed+Position+Activity+(1|FishID)	Speed	Medium - Slow	-0.022	0.018	207.69	-1.17	0.646	
Full data	SideBars ~ Speed+Position+Activity+(1|FishID)	Speed	Medium - Still	-0.088	0.022	197.47	-4.04	<0.001	***
Full data	SideBars ~ Speed+Position+Activity+(1|FishID)	Speed	Slow - Still	-0.066	0.022	193.38	-2.95	0.019	*
Full data	SideBars ~ Speed+Position+Activity+(1|FishID)	Position	Close - Far	0.065	0.023	217.15	2.86	0.005	**
Full data	SideBars ~ Speed+Position+Activity+(1|FishID)	Activity	aggression - cleaning	0.016	0.058	187.63	0.28	1	
Full data	SideBars ~ Speed+Position+Activity+(1|FishID)	Activity	aggression - hunting alone	-0.003	0.066	184.12	-0.05	1	
Full data	SideBars ~ Speed+Position+Activity+(1|FishID)	Activity	aggression - hunting with moray	0.061	0.085	199.74	0.71	0.98	
Full data	SideBars ~ Speed+Position+Activity+(1|FishID)	Activity	aggression - hunting with predators	0.011	0.065	194.45	0.17	1	
Full data	SideBars ~ Speed+Position+Activity+(1|FishID)	Activity	aggression - none	-0.018	0.051	170.75	-0.36	0.999	
Full data	SideBars ~ Speed+Position+Activity+(1|FishID)	Activity	cleaning - hunting alone	-0.019	0.053	219.04	-0.36	0.999	
Full data	SideBars ~ Speed+Position+Activity+(1|FishID)	Activity	cleaning - hunting with moray	0.045	0.074	143.29	0.6	0.991	
Full data	SideBars ~ Speed+Position+Activity+(1|FishID)	Activity	cleaning - hunting with predators	-0.005	0.052	231.65	-0.09	1	
Full data	SideBars ~ Speed+Position+Activity+(1|FishID)	Activity	cleaning - none	-0.034	0.03	227.65	-1.12	0.872	
Full data	SideBars ~ Speed+Position+Activity+(1|FishID)	Activity	hunting alone - hunting with moray	0.064	0.081	173.41	0.79	0.97	
Full data	SideBars ~ Speed+Position+Activity+(1|FishID)	Activity	hunting alone - hunting with predators	0.014	0.055	196.41	0.26	1	
Full data	SideBars ~ Speed+Position+Activity+(1|FishID)	Activity	hunting alone - none	-0.015	0.044	205.79	-0.34	0.999	
Full data	SideBars ~ Speed+Position+Activity+(1|FishID)	Activity	hunting with moray - hunting with predators	-0.05	0.081	164.25	-0.61	0.99	
Full data	SideBars ~ Speed+Position+Activity+(1|FishID)	Activity	hunting with moray - none	-0.079	0.069	121.37	-1.14	0.862	
Full data	SideBars ~	Activity	hunting with predators	-0.029	0.044	227.13	-0.67	0.985	

	Speed+Position+Activity+(1|FishID)		- none						
Full data	HeadStripe ~ Speed+Position+Activity+(1|FishID)	Speed	Fast - Medium	0.018	0.025	169.7	0.73	0.887	
Full data	HeadStripe ~ Speed+Position+Activity+(1|FishID)	Speed	Fast - Slow	-0.023	0.027	171.27	-0.85	0.831	
Full data	HeadStripe ~ Speed+Position+Activity+(1|FishID)	Speed	Fast - Still	-0.016	0.028	171.13	-0.59	0.936	
Full data	HeadStripe ~ Speed+Position+Activity+(1|FishID)	Speed	Medium - Slow	-0.041	0.015	173.58	-2.75	0.033	*
Full data	HeadStripe ~ Speed+Position+Activity+(1|FishID)	Speed	Medium - Still	-0.035	0.017	174	-1.99	0.194	
Full data	HeadStripe ~ Speed+Position+Activity+(1|FishID)	Speed	Slow - Still	0.006	0.018	172.79	0.36	0.984	
Full data	HeadStripe ~ Speed+Position+Activity+(1|FishID)	Position	Close - Far	-0.012	0.02	173.78	-0.6	0.546	
Full data	HeadStripe ~ Speed+Position+Activity+(1|FishID)	Activity	aggression - cleaning	0.177	0.044	173.83	4.06	0.001	**
Full data	HeadStripe ~ Speed+Position+Activity+(1|FishID)	Activity	aggression - hunting alone	0.185	0.051	173.65	3.63	0.005	**
Full data	HeadStripe ~ Speed+Position+Activity+(1|FishID)	Activity	aggression - hunting with moray	0.179	0.051	108.31	3.48	0.009	**
Full data	HeadStripe ~ Speed+Position+Activity+(1|FishID)	Activity	aggression - hunting with predators	-0.171	0.047	173.95	-3.67	0.004	**
Full data	HeadStripe ~ Speed+Position+Activity+(1|FishID)	Activity	aggression - none	0.177	0.038	172.62	4.7	<0.001	***
Full data	HeadStripe ~ Speed+Position+Activity+(1|FishID)	Activity	cleaning - hunting alone	0.008	0.042	155.84	0.2	1	
Full data	HeadStripe ~ Speed+Position+Activity+(1|FishID)	Activity	cleaning - hunting with moray	0.003	0.043	76.01	0.06	1	
Full data	HeadStripe ~ Speed+Position+Activity+(1|FishID)	Activity	cleaning - hunting with predators	-0.348	0.037	148.85	-9.51	<0.001	***
Full data	HeadStripe ~ Speed+Position+Activity+(1|FishID)	Activity	cleaning - none	0.001	0.024	173.82	0.03	1	
Full data	HeadStripe ~ Speed+Position+Activity+(1|FishID)	Activity	hunting alone - hunting with moray	-0.006	0.05	88.44	-0.12	1	
Full data	HeadStripe ~ Speed+Position+Activity+(1|FishID)	Activity	hunting alone - hunting with predators	-0.357	0.044	173.81	-8.18	<0.001	***
Full data	HeadStripe ~ Speed+Position+Activity+(1|FishID)	Activity	hunting alone - none	-0.008	0.036	159.94	-0.21	1	
Full data	HeadStripe ~ Speed+Position+Activity+(1|FishID)	Activity	hunting with moray - hunting with predators	-0.351	0.046	73.51	-7.63	<0.001	***
Full data	HeadStripe ~ Speed+Position+Activity+(1|FishID)	Activity	hunting with moray - none	-0.002	0.036	50	-0.05	1	
Full data	HeadStripe ~ Speed+Position+Activity+(1|FishID)	Activity	hunting with predators - none	0.349	0.03	145.17	11.73	<0.001	***

Only "none" activity	BodyColor ~ Speed+Position+(1|FishID)	Speed	Fast - Medium	0.032	0.018	160.67	1.84	0.26	
Only "none" activity	BodyColor ~ Speed+Position+(1|FishID)	Speed	Fast - Slow	0.053	0.019	155.85	2.87	0.024	*
Only "none" activity	BodyColor ~ Speed+Position+(1|FishID)	Speed	Fast - Still	0.055	0.02	152.06	2.76	0.033	*
Only "none" activity	BodyColor ~ Speed+Position+(1|FishID)	Speed	Medium - Slow	0.021	0.013	169.47	1.65	0.352	
Only "none" activity	BodyColor ~ Speed+Position+(1|FishID)	Speed	Medium - Still	0.023	0.015	159.08	1.57	0.399	
Only "none" activity	BodyColor ~ Speed+Position+(1|FishID)	Speed	Slow - Still	0.002	0.015	151.4	0.12	0.999	
Only "none" activity	BodyColor ~ Speed+Position+(1|FishID)	Position	Close - Far	-0.051	0.015	175.77	-3.51	0.001	***
Only "none" activity	BodyPatches ~ Speed+Position+(1|FishID)	Speed	Fast - Medium	0.003	0.027	184.18	0.11	1	
Only "none" activity	BodyPatches ~ Speed+Position+(1|FishID)	Speed	Fast - Slow	-0.049	0.029	182.69	-1.67	0.345	
Only "none" activity	BodyPatches ~ Speed+Position+(1|FishID)	Speed	Fast - Still	-0.284	0.032	181.09	-9	<0.001	***
Only "none" activity	BodyPatches ~ Speed+Position+(1|FishID)	Speed	Medium - Slow	-0.052	0.02	186.8	-2.62	0.047	*
Only "none" activity	BodyPatches ~ Speed+Position+(1|FishID)	Speed	Medium - Still	-0.287	0.023	183.96	-12.53	<0.001	***
Only "none" activity	BodyPatches ~ Speed+Position+(1|FishID)	Speed	Slow - Still	-0.235	0.024	177.95	-9.73	<0.001	***
Only "none" activity	BodyPatches ~ Speed+Position+(1|FishID)	Position	Close - Far	0.012	0.022	193.15	0.56	0.576	
Only "none" activity	SideBars ~ Speed+Position+(1|FishID)	Speed	Fast - Medium	0.001	0.028	162.91	0.05	1	
Only "none" activity	SideBars ~ Speed+Position+(1|FishID)	Speed	Fast - Slow	-0.031	0.029	158.32	-1.05	0.72	
Only "none" activity	SideBars ~ Speed+Position+(1|FishID)	Speed	Fast - Still	-0.108	0.031	154.82	-3.42	0.004	**
Only "none" activity	SideBars ~ Speed+Position+(1|FishID)	Speed	Medium - Slow	-0.032	0.02	171.27	-1.61	0.378	
Only "none" activity	SideBars ~ Speed+Position+(1|FishID)	Speed	Medium - Still	-0.109	0.023	161.69	-4.73	<0.001	***
Only "none" activity	SideBars ~ Speed+Position+(1|FishID)	Speed	Slow - Still	-0.077	0.024	153.86	-3.21	0.009	**
Only "none" activity	SideBars ~ Speed+Position+(1|FishID)	Position	Close - Far	0.067	0.023	181.96	2.92	0.004	**
Only "none" activity	HeadStripe ~ Speed+Position+(1|FishID)	Speed	Fast - Medium	<0.001	0.01	137.81	<0.01	1	
Only "none"	HeadStripe ~	Speed	Fast - Slow	-0.002	0.01	136.63	-0.17	0.998	

activity	Speed+Position+(1|FishID)								
Only "none" activity	HeadStripe ~ Speed+Position+(1|FishID)	Speed	Fast - Still	-0.012	0.011	137.12	-1.16	0.653	
Only "none" activity	HeadStripe ~ Speed+Position+(1|FishID)	Speed	Medium - Slow	-0.002	0.006	139.75	-0.29	0.992	
Only "none" activity	HeadStripe ~ Speed+Position+(1|FishID)	Speed	Medium - Still	-0.012	0.007	139.91	-1.82	0.271	
Only "none" activity	HeadStripe ~ Speed+Position+(1|FishID)	Speed	Slow - Still	-0.011	0.007	138.5	-1.5	0.442	
Only "none" activity	HeadStripe ~ Speed+Position+(1|FishID)	Position	Close - Far	<0.001	0.008	138.12	0.06	0.949	
No "fast" speed, No "far" Position	BodyColor ~ Speed+Activity+(1|FishID)	Speed	Medium - Slow	0.004	0.012	166.98	0.3	0.951	
No "fast" speed, No "far" Position	BodyColor ~ Speed+Activity+(1|FishID)	Speed	Medium - Still	0.016	0.014	163.79	1.12	0.5	
No "fast" speed, No "far" Position	BodyColor ~ Speed+Activity+(1|FishID)	Speed	Slow - Still	0.012	0.015	156.66	0.84	0.681	
No "fast" speed, No "far" Position	BodyColor ~ Speed+Activity+(1|FishID)	Activity	aggression - cleaning	0.041	0.037	149.46	1.1	0.882	
No "fast" speed, No "far" Position	BodyColor ~ Speed+Activity+(1|FishID)	Activity	aggression - hunting alone	0.006	0.042	142.01	0.13	1	
No "fast" speed, No "far" Position	BodyColor ~ Speed+Activity+(1|FishID)	Activity	aggression - hunting with moray	-0.053	0.056	110.3	-0.93	0.937	
No "fast" speed, No "far" Position	BodyColor ~ Speed+Activity+(1|FishID)	Activity	aggression - hunting with predators	0.011	0.042	153.18	0.28	1	
No "fast" speed, No "far" Position	BodyColor ~ Speed+Activity+(1|FishID)	Activity	aggression - none	0.028	0.032	130.32	0.86	0.956	
No "fast" speed, No "far" Position	BodyColor ~ Speed+Activity+(1|FishID)	Activity	cleaning - hunting alone	-0.035	0.034	178.7	-1.03	0.906	
No "fast" speed, No "far" Position	BodyColor ~ Speed+Activity+(1|FishID)	Activity	cleaning - hunting with moray	-0.094	0.05	69.48	-1.86	0.432	
No "fast" speed, No "far" Position	BodyColor ~ Speed+Activity+(1|FishID)	Activity	cleaning - hunting with predators	-0.03	0.034	187.45	-0.86	0.955	
No "fast" speed, No "far" Position	BodyColor ~ Speed+Activity+(1|FishID)	Activity	cleaning - none	-0.013	0.021	186.99	-0.64	0.987	
No "fast" speed, No "far" Position	BodyColor ~ Speed+Activity+(1|FishID)	Activity	hunting alone - hunting with moray	-0.058	0.054	90.43	-1.07	0.89	
No "fast" speed, No "far" Position	BodyColor ~ Speed+Activity+(1|FishID)	Activity	hunting alone - hunting with predators	0.006	0.035	151.09	0.17	1	
No "fast" speed, No "far" Position	BodyColor ~ Speed+Activity+(1|FishID)	Activity	hunting alone - none	0.022	0.029	164.5	0.78	0.971	
No "fast" speed, No "far" Position	BodyColor ~ Speed+Activity+(1|FishID)	Activity	hunting with moray - hunting with predators	0.064	0.054	84.65	1.19	0.842	
No "fast" speed, No "far" Position	BodyColor ~ Speed+Activity+(1|FishID)	Activity	hunting with moray - none	0.08	0.047	56.55	1.72	0.524	

No "fast" speed, No "far" Position	BodyColor ~ Speed+Activity+(1|FishID)	Activity	hunting with predators - none	0.016	0.028	184.67	0.57	0.993	
No "fast" speed, No "far" Position	BodyPatches ~ Speed+Activity+(1|FishID)	Speed	Medium - Slow	-0.055	0.021	176.25	-2.68	0.022	*
No "fast" speed, No "far" Position	BodyPatches ~ Speed+Activity+(1|FishID)	Speed	Medium - Still	-0.268	0.023	173.96	-11.44	<0.001	***
No "fast" speed, No "far" Position	BodyPatches ~ Speed+Activity+(1|FishID)	Speed	Slow - Still	-0.213	0.024	168.2	-8.74	<0.001	***
No "fast" speed, No "far" Position	BodyPatches ~ Speed+Activity+(1|FishID)	Activity	aggression - cleaning	-0.004	0.068	169.49	-0.06	1	
No "fast" speed, No "far" Position	BodyPatches ~ Speed+Activity+(1|FishID)	Activity	aggression - hunting alone	0.07	0.078	176.42	0.9	0.947	
No "fast" speed, No "far" Position	BodyPatches ~ Speed+Activity+(1|FishID)	Activity	aggression - hunting with moray	0.137	0.087	126.88	1.57	0.618	
No "fast" speed, No "far" Position	BodyPatches ~ Speed+Activity+(1|FishID)	Activity	aggression - hunting with predators	-0.058	0.078	183.22	-0.74	0.976	
No "fast" speed, No "far" Position	BodyPatches ~ Speed+Activity+(1|FishID)	Activity	aggression - none	-0.009	0.062	162.45	-0.15	1	
No "fast" speed, No "far" Position	BodyPatches ~ Speed+Activity+(1|FishID)	Activity	cleaning - hunting alone	0.073	0.056	183.46	1.32	0.775	
No "fast" speed, No "far" Position	BodyPatches ~ Speed+Activity+(1|FishID)	Activity	cleaning - hunting with moray	0.141	0.069	67.07	2.04	0.332	
No "fast" speed, No "far" Position	BodyPatches ~ Speed+Activity+(1|FishID)	Activity	cleaning - hunting with predators	-0.054	0.057	167.21	-0.95	0.931	
No "fast" speed, No "far" Position	BodyPatches ~ Speed+Activity+(1|FishID)	Activity	cleaning - none	-0.005	0.033	182.76	-0.17	1	
No "fast" speed, No "far" Position	BodyPatches ~ Speed+Activity+(1|FishID)	Activity	hunting alone - hunting with moray	0.067	0.077	89.22	0.87	0.952	
No "fast" speed, No "far" Position	BodyPatches ~ Speed+Activity+(1|FishID)	Activity	hunting alone - hunting with predators	-0.128	0.06	162.64	-2.12	0.282	
No "fast" speed, No "far" Position	BodyPatches ~ Speed+Activity+(1|FishID)	Activity	hunting alone - none	-0.079	0.047	183.2	-1.67	0.553	
No "fast" speed, No "far" Position	BodyPatches ~ Speed+Activity+(1|FishID)	Activity	hunting with moray - hunting with predators	-0.195	0.078	80.33	-2.5	0.137	
No "fast" speed, No "far" Position	BodyPatches ~ Speed+Activity+(1|FishID)	Activity	hunting with moray - none	-0.146	0.063	51.44	-2.34	0.197	
No "fast" speed, No "far" Position	BodyPatches ~ Speed+Activity+(1|FishID)	Activity	hunting with predators - none	0.049	0.049	168.03	1	0.916	
No "fast" speed, No "far" Position	SideBars ~ Speed+Activity+(1|FishID)	Speed	Medium - Slow	-0.021	0.021	164.57	-1	0.579	
No "fast" speed, No "far" Position	SideBars ~ Speed+Activity+(1|FishID)	Speed	Medium - Still	-0.093	0.024	161.31	-3.89	<0.001	***
No "fast" speed, No "far" Position	SideBars ~ Speed+Activity+(1|FishID)	Speed	Slow - Still	-0.072	0.025	154.68	-2.93	0.011	*
No "fast" speed,	SideBars ~	Activity	aggression - cleaning	0.021	0.063	147.44	0.33	0.999	

No "far" Position	Speed+Activity+(1|FishID)								
No "fast" speed, No "far" Position	SideBars ~ Speed+Activity+(1|FishID)	Activity	aggression - hunting alone	-0.001	0.07	139.93	-0.02	1	
No "fast" speed, No "far" Position	SideBars ~ Speed+Activity+(1|FishID)	Activity	aggression - hunting with moray	0.067	0.096	108.3	0.69	0.982	
No "fast" speed, No "far" Position	SideBars ~ Speed+Activity+(1|FishID)	Activity	aggression - hunting with predators	0.015	0.07	151.02	0.21	1	
No "fast" speed, No "far" Position	SideBars ~ Speed+Activity+(1|FishID)	Activity	aggression - none	-0.022	0.054	128.35	-0.41	0.998	
No "fast" speed, No "far" Position	SideBars ~ Speed+Activity+(1|FishID)	Activity	cleaning - hunting alone	-0.022	0.058	176.46	-0.39	0.999	
No "fast" speed, No "far" Position	SideBars ~ Speed+Activity+(1|FishID)	Activity	cleaning - hunting with moray	0.046	0.086	69.33	0.53	0.995	
No "fast" speed, No "far" Position	SideBars ~ Speed+Activity+(1|FishID)	Activity	cleaning - hunting with predators	-0.006	0.057	185.99	-0.1	1	
No "fast" speed, No "far" Position	SideBars ~ Speed+Activity+(1|FishID)	Activity	cleaning - none	-0.043	0.035	185.53	-1.25	0.811	
No "fast" speed, No "far" Position	SideBars ~ Speed+Activity+(1|FishID)	Activity	hunting alone - hunting with moray	0.068	0.092	89.52	0.74	0.977	
No "fast" speed, No "far" Position	SideBars ~ Speed+Activity+(1|FishID)	Activity	hunting alone - hunting with predators	0.016	0.059	149.33	0.28	1	
No "fast" speed, No "far" Position	SideBars ~ Speed+Activity+(1|FishID)	Activity	hunting alone - none	-0.021	0.048	162	-0.44	0.998	
No "fast" speed, No "far" Position	SideBars ~ Speed+Activity+(1|FishID)	Activity	hunting with moray - hunting with predators	-0.052	0.092	84.18	-0.56	0.993	
No "fast" speed, No "far" Position	SideBars ~ Speed+Activity+(1|FishID)	Activity	hunting with moray - none	-0.089	0.08	56.79	-1.11	0.874	
No "fast" speed, No "far" Position	SideBars ~ Speed+Activity+(1|FishID)	Activity	hunting with predators - none	-0.037	0.048	182.69	-0.78	0.97	
No "fast" speed, No "far" Position	HeadStripe ~ Speed+Activity+(1|FishID)	Speed	Medium - Slow	-0.044	0.017	145.3	-2.6	0.027	*
No "fast" speed, No "far" Position	HeadStripe ~ Speed+Activity+(1|FishID)	Speed	Medium - Still	-0.036	0.019	145.97	-1.89	0.146	
No "fast" speed, No "far" Position	HeadStripe ~ Speed+Activity+(1|FishID)	Speed	Slow - Still	0.008	0.02	144.46	0.39	0.918	
No "fast" speed, No "far" Position	HeadStripe ~ Speed+Activity+(1|FishID)	Activity	aggression - cleaning	0.177	0.048	145.76	3.67	0.005	**
No "fast" speed, No "far" Position	HeadStripe ~ Speed+Activity+(1|FishID)	Activity	aggression - hunting alone	0.187	0.056	145.09	3.35	0.013	*
No "fast" speed, No "far" Position	HeadStripe ~ Speed+Activity+(1|FishID)	Activity	aggression - hunting with moray	0.179	0.059	80.21	3.03	0.037	*
No "fast" speed, No "far" Position	HeadStripe ~ Speed+Activity+(1|FishID)	Activity	aggression - hunting with predators	-0.172	0.051	145.98	-3.36	0.012	*
No "fast" speed, No "far" Position	HeadStripe ~ Speed+Activity+(1|FishID)	Activity	aggression - none	0.178	0.041	143.76	4.28	<0.001	***

No "fast" speed, No "far" Position	HeadStripe ~ Speed+Activity+(1|FishID)	Activity	cleaning - hunting alone	0.01	0.047	130.12	0.21	1	
No "fast" speed, No "far" Position	HeadStripe ~ Speed+Activity+(1|FishID)	Activity	cleaning - hunting with moray	0.002	0.05	56.07	0.04	1	
No "fast" speed, No "far" Position	HeadStripe ~ Speed+Activity+(1|FishID)	Activity	cleaning - hunting with predators	-0.349	0.041	119.47	-8.56	<0.001	***
No "fast" speed, No "far" Position	HeadStripe ~ Speed+Activity+(1|FishID)	Activity	cleaning - none	<0.001	0.028	144.73	0.01	1	
No "fast" speed, No "far" Position	HeadStripe ~ Speed+Activity+(1|FishID)	Activity	hunting alone - hunting with moray	-0.008	0.058	65.98	-0.13	1	
No "fast" speed, No "far" Position	HeadStripe ~ Speed+Activity+(1|FishID)	Activity	hunting alone - hunting with predators	-0.359	0.048	145.75	-7.52	<0.001	***
No "fast" speed, No "far" Position	HeadStripe ~ Speed+Activity+(1|FishID)	Activity	hunting alone - none	-0.009	0.04	135.81	-0.24	1	
No "fast" speed, No "far" Position	HeadStripe ~ Speed+Activity+(1|FishID)	Activity	hunting with moray - hunting with predators	-0.351	0.053	54.7	-6.58	<0.001	***
No "fast" speed, No "far" Position	HeadStripe ~ Speed+Activity+(1|FishID)	Activity	hunting with moray - none	-0.002	0.044	38.09	-0.04	1	
No "fast" speed, No "far" Position	HeadStripe ~ Speed+Activity+(1|FishID)	Activity	hunting with predators - none	0.35	0.033	115.97	10.69	<0.001	***

Table S7: ANOVA analysis of the pattern type effects on the total magnitude of their changes, in events that lasted up to 15 seconds. (TotalChange ~ Pattern)

Significance codes: '***'0.001, '**'0.01, '*'0.05

Term	DF	Sum Sq	Mean Sq	F value	Pr(>F)	Significance Codes
Pattern	2	144842	72421	137.4	<0.001	***
Residuals	180	94857	527			

Table S8: ANOVA analysis of the pattern type effects on the rate of their changes, in events that lasted up to 15 seconds. (ChangeRate ~ Pattern).

Significance codes: '***'0.001, '**'0.01, '*'0.05

Term	DF	Sum Sq	Mean Sq	F value	Pr(>F)	Significance Codes
Pattern	2	4937	2468	46.77	<0.001	***
Residuals	180	9500	52.78			

Table S9: Response of each color channel of the body color to the behavioral effects.

Fit: manova((BodyColor_R, BodyColor_G, BodyColor_B) ~ Speed + Position + Activity)

Significance codes: '***'0.001, '**'0.01, '*'0.05

Term	DF	Sum Sq	Mean Sq	F value	Pr(>F)	Sig.	
Speed	3	0.017	0.006	1.874	0.134492311		**Red**
Position	1	0.030	0.030	9.874	0.001882834	**	
Activity	5	0.019	0.004	1.255	0.284072228		
Residuals	245	0.746	0.003				
Speed	3	0.132	0.044	4.386	0.004989471	**	**Green**
Position	1	0.153	0.153	15.245	0.000122118	***	
Activity	5	0.059	0.012	1.173	0.323002276		
Residuals	245	2.452	0.010				
Speed	3	0.161	0.054	6.072	0.000530274	***	**Blue**
Position	1	0.152	0.152	17.130	4.80391E-05	***	
Activity	5	0.068	0.014	1.535	0.179414067		
Residuals	245	2.168	0.009				
